# Supplementary figures and images for: Mining host candidate regulators of schistosomiasis-induced liver fibrosis in response to artesunate therapy through transcriptomics approach
Source: PLoS Negl Trop Dis. 2023 Sep 29;17(9):e0011626. doi: 10.1371/journal.pntd.0011626 (PMC10566724; doi:10.1371/journal.pntd.0011626)

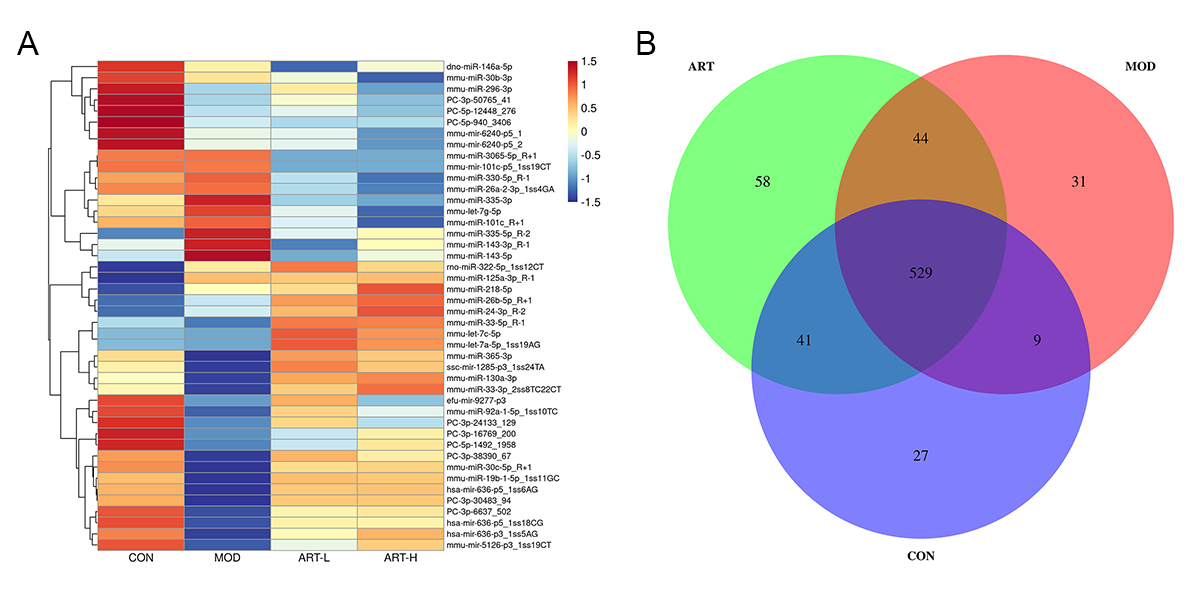

Supplement: S1 Fig — (A) Heat map of differentially expressed miRNAs and (B) Venn diagram of differentially expressed miRNAs. (TIF) [file pntd.0011626.s001.tif]
