# Supplementary material for: Mining host candidate regulators of schistosomiasis-induced liver fibrosis in response to artesunate therapy through transcriptomics approach
Source: PLoS Negl Trop Dis. 2023 Sep 29;17(9):e0011626. doi: 10.1371/journal.pntd.0011626 (PMC10566724; doi:10.1371/journal.pntd.0011626)
Supplement: S1 Table — (DOC) [file pntd.0011626.s002.doc]

| **Gene ID** | **Gene name** | **Sequence (5’→3’)** | **Length (bp)** |
| --- | --- | --- | --- |
| XM_021629767.1 | Ctss | GAGGCTTTCCAGTACGTCATCG | 280 |
|  |  | CACCGTGGTTCACCTTGTCAGT |  |
| M_021644282.1 | Mgp | GCAGCCCTGTGCTACGAATCT | 249 |
|  |  | CTGCCTGAAGTAGCGGTTGTAG |  |
| XM_021627622.1 | Vim | TGTCCGCCAGCAGTATGAAAGT | 215 |
|  |  | GGCGTTCCAGGGACTCGTTA |  |
| XM_021626690.1 | Ctgf | ATCTCCACCCGTGTTACCAATG | 158 |
|  |  | TCGAACTTGACAGGCTTGGTGA |  |
| XM_021633714.1 | Cd74 | CTTCCTAAATCTGCCAAACCTGT | 329 |
|  |  | GGCAGAGTCTCAGTGGGCTT |  |
| XM_021633803.1 | Chi3l1 | CACCACCCTCATCAAGGAACTA | 298 |
|  |  | GGCTCCCAGTCTCAGCATGTAC |  |
| M_021636934.1 | GAPDH | CCATCACTGCTACCCAGAAGACC | 288 |
|  |  | AGCCCAGGATGCCCTTTAGTG |  |
