# Supplementary material for: Mining host candidate regulators of schistosomiasis-induced liver fibrosis in response to artesunate therapy through transcriptomics approach
Source: PLoS Negl Trop Dis. 2023 Sep 29;17(9):e0011626. doi: 10.1371/journal.pntd.0011626 (PMC10566724; doi:10.1371/journal.pntd.0011626)
Supplement: S2 Table — (DOC) [file pntd.0011626.s003.doc]

| Group  Group (n=4, means ± SD) | Liver mass index（mg/g） |
| --- | --- |
|  | Liver |
| CON | 32.32 ± 2.32 |
| MOD | 38.88 ± 7.37 |
| ART-L | 37.26 ± 3.15 |
| ART-H | 31.86 ± 2.69 |
