# Supplementary material for: Mining host candidate regulators of schistosomiasis-induced liver fibrosis in response to artesunate therapy through transcriptomics approach
Source: PLoS Negl Trop Dis. 2023 Sep 29;17(9):e0011626. doi: 10.1371/journal.pntd.0011626 (PMC10566724; doi:10.1371/journal.pntd.0011626)
Supplement: S3 Table — (DOC) [file pntd.0011626.s004.doc]

| **Sample** | **Raw Data** | | **Valid Data** | | **Valid Ratio(reads)** | **Q20%** | **Q30%** | **GC content%** |
| --- | --- | --- | --- | --- | --- | --- | --- | --- |
|  | **Read** | **Base** | **Read** | **Base** |  |  |  |  |
| CON_1 | 9E+07 | 13.74G | 9E+07 | 13.36G | 97.24 | 99.58 | 96.67 | 49 |
| CON_2 | 9E+07 | 13.79G | 9E+07 | 13.19G | 95.65 | 99.54 | 96.87 | 49 |
| MOD_1 | 9E+07 | 13.92G | 9E+07 | 13.38G | 96.11 | 99.60 | 96.80 | 49 |
| MOD_2 | 9E+07 | 13.54G | 9E+07 | 13.11G | 96.82 | 99.78 | 97.46 | 49 |
| ART_L | 9E+07 | 13.96G | 9E+07 | 13.50G | 96.70 | 99.78 | 97.65 | 49 |
| ART_H | 7E+07 | 10.55G | 7E+07 | 10.24G | 97.08 | 99.83 | 97.78 | 48 |
