# Supplementary material for: Mining host candidate regulators of schistosomiasis-induced liver fibrosis in response to artesunate therapy through transcriptomics approach
Source: PLoS Negl Trop Dis. 2023 Sep 29;17(9):e0011626. doi: 10.1371/journal.pntd.0011626 (PMC10566724; doi:10.1371/journal.pntd.0011626)
Supplement: S4 Table — (DOC) [file pntd.0011626.s005.doc]

| **Sample** | **Valid reads** | **Mapped reads** | **Unique Mapped reads** |
| --- | --- | --- | --- |
| CON_1 | 84694392 | 78243791(92.38%) | 54296230(64.11%) |
| CON_2 | 83424602 | 77585142(93.00%) | 54866794(65.77%) |
| MOD_1 | 84769906 | 78598668(92.72%) | 54742666(64.58%) |
| MOD_2 | 84220350 | 77308980(91.79%) | 55525870(65.93%) |
| ART_L | 85726578 | 79972246(93.29%) | 57030442(66.53%) |
| ART_H | 64215940 | 59799312(93.12%) | 43766561(68.16%) |
