# Supplementary material for: Mining host candidate regulators of schistosomiasis-induced liver fibrosis in response to artesunate therapy through transcriptomics approach
Source: PLoS Negl Trop Dis. 2023 Sep 29;17(9):e0011626. doi: 10.1371/journal.pntd.0011626 (PMC10566724; doi:10.1371/journal.pntd.0011626)
Supplement: S5 Table — (DOCX) [file pntd.0011626.s006.docx]

| **miRNA ID** | **Transcript ID** | **Gene ID** | **Symbol** | **Gene Annoation** | **TargetScan score** | **miranda Energy** |
| --- | --- | --- | --- | --- | --- | --- |
| miR-193a-3p | XM_021635227.1 | 110547587 | Prkcd | protein kinase C delta | 54 | -20.67 |
|  | XM_021635228.1 | 110547587 | Prkcd | protein kinase C delta | 54 | -20.67 |
|  | XM_021662989.1 | 110565332 | Tlr4 | toll like receptor 4 | 83 | -17.24 |
| miR-2137 | XM_021652085.1 | 110557597 | Nckap1l | NCK associated protein 1 like | 66 | -23.84 |
| miR-3473b | XM_021651946.1 | 110557518 | Adamtsl2 | ADAMTS like 2 | 76 | -21.6 |
|  | XM_021643451.1 | 110552284 | Ddit4 | DNA damage inducible transcript 4 | 70 | -23.82 |
|  | XM_021652968.1 | 110558185 | Fscn1 | fascin actin-bundling protein 1 | 95 | -24.36 |
|  | XM_021652871.1 | 110558118 | Myo1f | myosin IF | 54 | -23.33 |
|  | XM_021652872.1 | 110558118 | Myo1f | myosin IF | 51 | -23.33 |
|  | XM_021652873.1 | 110558118 | Myo1f | myosin IF | 51 | -23.33 |
|  | XM_021664050.1 | 110566227 | Src | SRC proto-oncogene, non-receptor tyrosine kinase | 87 | -21.77 |
|  | XM_021664053.1 | 110566227 | Src | SRC proto-oncogene, non-receptor tyrosine kinase | 87 | -21.77 |
|  | XM_021664061.1 | 110566227 | Src | SRC proto-oncogene, non-receptor tyrosine kinase | 87 | -21.77 |
|  | XM_021664070.1 | 110566227 | Src | SRC proto-oncogene, non-receptor tyrosine kinase | 87 | -21.77 |
|  | XM_021664076.1 | 110566227 | Src | SRC proto-oncogene, non-receptor tyrosine kinase | 87 | -21.77 |
|  | XM_021651936.1 | 110557509 | Surf4 | surfeit 4 | 93 | -15.27 |
| miR-3473e | XM_021651946.1 | 110557518 | Adamtsl2 | ADAMTS like 2 | 76 | -21.6 |
|  | XM_021643451.1 | 110552284 | Ddit4 | DNA damage inducible transcript 4 | 70 | -23.82 |
|  | XM_021652968.1 | 110558185 | Fscn1 | fascin actin-bundling protein 1 | 95 | -24.36 |
|  | XM_021652871.1 | 110558118 | Myo1f | myosin IF | 54 | -23.33 |
|  | XM_021652872.1 | 110558118 | Myo1f | myosin IF | 51 | -23.33 |
|  | XM_021652873.1 | 110558118 | Myo1f | myosin IF | 51 | -23.33 |
|  | XM_021664050.1 | 110566227 | Src | SRC proto-oncogene, non-receptor tyrosine kinase | 87 | -21.77 |
|  | XM_021664053.1 | 110566227 | Src | SRC proto-oncogene, non-receptor tyrosine kinase | 87 | -21.77 |
|  | XM_021664061.1 | 110566227 | Src | SRC proto-oncogene, non-receptor tyrosine kinase | 87 | -21.77 |
|  | XM_021664070.1 | 110566227 | Src | SRC proto-oncogene, non-receptor tyrosine kinase | 87 | -21.77 |
|  | XM_021664076.1 | 110566227 | Src | SRC proto-oncogene, non-receptor tyrosine kinase | 87 | -21.77 |
|  | XM_021651936.1 | 110557509 | Surf4 | surfeit 4 | 93 | -15.27 |
| miR-365-3p | XM_021636713.1 | 110548451 | Cyth4 | cytohesin 4 | 67 | -12.24 |
|  | XM_021636714.1 | 110548451 | Cyth4 | cytohesin 4 | 65 | -12.24 |
|  | XM_021636715.1 | 110548451 | Cyth4 | cytohesin 4 | 67 | -12.24 |
|  | XM_021636716.1 | 110548451 | Cyth4 | cytohesin 4 | 67 | -12.24 |
| miR-370-3p | XM_021636713.1 | 110548451 | Cyth4 | cytohesin 4 | 62 | -24.92 |
|  | XM_021636714.1 | 110548451 | Cyth4 | cytohesin 4 | 62 | -24.92 |
|  | XM_021636715.1 | 110548451 | Cyth4 | cytohesin 4 | 62 | -24.92 |
|  | XM_021636716.1 | 110548451 | Cyth4 | cytohesin 4 | 62 | -24.92 |
|  | XM_021643451.1 | 110552284 | Ddit4 | DNA damage inducible transcript 4 | 67 | -23.41 |
|  | XM_021641529.1 | 110551150 | Irf8 | interferon regulatory factor 8 | 78 | -50.31 |
