# Supplementary material for: Mining host candidate regulators of schistosomiasis-induced liver fibrosis in response to artesunate therapy through transcriptomics approach
Source: PLoS Negl Trop Dis. 2023 Sep 29;17(9):e0011626. doi: 10.1371/journal.pntd.0011626 (PMC10566724; doi:10.1371/journal.pntd.0011626)
Supplement: Attachment — Submitted filename: Regulators of Schistosomiasis Liver Fibrosis to Artesunate.pdf [file pntd.0011626.s007.pdf]

Mining Host Candidate Regulators of Schistosomiasis Liver Fibrosis in Response to Artesunate Therapy through Transcriptomics Approach

Yajie Yuan[1], Xinyue Lv[1], Fangwei Dai[2], Haojie Ding[3], Riping Chen[4], Bin Zheng[3], Wenxia Zhao[4], Qunbo Tong[3], Jianzu Ding[3], Di Lou[3], Xiaofeng Chu[2], Shaohong Lu[3*] and Qingming Kong[1,3*]

[1] School of Laboratory Medicine and Bioengineering, Hangzhou Medical College (Zhejiang Academy of Medical Sciences), Hangzhou, China

[2]Zhejiang Key Laboratory of Experimental Animal and Safety Evaluation, Hangzhou Medical College (Zhejiang Academy of Medical Sciences), Hangzhou, China

[3]Institute of Parasitic Diseases, Hangzhou Medical College (Zhejiang Academy of Medical Sciences), Hangzhou, China

[4]School of Public Health, Hangzhou Medical College (Zhejiang Academy of Medical Sciences), Hangzhou, China

* **Correspondence:**
Shaohong Lu
llsshh2003@163.com
Qingming Kong
qmkong_1025@163.com

Abstract

Background: Artesunate (ART), has been reported to have an anti-fibrosis effect in various organs, yet the mechanism has not been systematically elucidated. **Aim**: This study is to clarify the effect and possible mechanisms of ART against liver fibrosis in Mongolian gerbil model induced by *Schistosoma japonicum* (*S. japonicum*). **Methods**: The effect of ART on hepatic stellate cells (HSCs) was measured by CCK-8 and Annexin V-FITC/PI staining assays. Liver fibrosis experimental model was established in Mongolian gerbil infected with Schistosoma japonicum cercariae and administered with praziquantel (PZQ), and then treated with 20 mg/kg or 40 mg/kg ART. Biochemical indicators (HA, ALT, GDA and GPX) levels were measured and histopathological changes of liver tissues were observed before and after ART treatment. Whole-transcriptome RNA sequencing (RNA-Seq) of the liver tissues was performed. Differentially expressed genes (DEGs) were identified by bioinformation analysis and verified by qPCR. **Results**: ART significantly inhibited the proliferation and induce apoptosis of HSCs in a dose-dependent manner. In vivo, GPX activity was significantly higher in the ART-H group than that of in the MOD group, and collagen was decreased significantly compared to that of before administration ($p<0.05$). We identified 158 DEGs and 131 differentially expressed miRNAs related to ART anti-schistosomiasis liver fibrosis. The qPCR results of selected DEGs are consistent with the sequencing results. These DEGs implicate central pathways, such as immune and inflammatory response and integrin-mediated signaling pathway et al. **Conclusion:** We developed liver fibrosis model in Mongolian gerbil mimicking human infection with *S. japonicum* cercariae. The efficacy test showed that ART is effective in preventing liver fibrosis in vitro and in infectious animal model. ART may alleviate effectively schistosomiasis liver fibrosis by decreasing inflammation and collagen deposition. We established the first systematically transcription profile and mined host candidate regulators of Schistosomiasis Liver Fibrosis in response to ART.

Keywords: Schistosoma, infectious animal model, liver fibrosis, hepatic stellate cells, artesunate.

Introduction

Liver fibrosis is a very complex dynamic pathological process including hepatic stellate cells (HSCs) activation, immunity and inflammation, increased extracellular matrix (ECM) proteins and fibrogenesis, in which involved the activation or inhibition of multiple signaling pathways in various cell types [1]. The major risk factors for liver fibrosis include hepatitis virus infection, *S. japonicum* infection, alcoholic liver disease, drugs and metabolic disorders [2]. Numerous researches on the mechanism of liver fibrosis have focused mainly on the HSCs activation and thus activated HSCs has been considered to be antifibrotic target [3]. Many drugs or small molecule are in clinical trials but less have been approved for the treatment of

liver fibrosis in clinical [4].

Dozens of natural compounds demonstrate antifibrotic activities in various liver fibrosis model in vivo and HSCs in vitro. Fuzheng Huayu (FZHY) can attenuate liver fibrosis by inhibiting HSCs activation and inflammatory response, regulation of ECM deposition, inhibition of hepatic sinusoidal capillarity and angiogenesis, in consequence, it has been approved by China food and drug administration for the treatment of liver fibrosis [5, 6]. Recently, Luo et al showed FZHY and ART could alleviate schistosomiasis liver fibrosis by modulating mitochondrial autophagy [7]. ART, a stable derivative of artemisinin extracted from the stems and leaves of artemisia annua, has many pharmacological effects including anti-inflammatory, anti-tumor and anti-fibrosis activities [8-10]. Additionally, ART can inhibit fibrosis through different mechanisms involving inflammatory infiltration, autophagy of ferritin, collagen synthesis and HSCs apoptosis etc. [11-13]. Several studies also proved that ART prevent schistosomiasis liver fibrosis by regulating mitochondrial function [7, 14]. At present, existing studies about anti-fibrosis mechanism of ART remain fragmented ~~and unsystematic~~.

Mongolian gerbils are relatively suitable hosts for Schistosoma japonicum. The schistosomiasis hepatic fibrosis Mongolian gerbil model has stable fibrosis formation for the eggs continued slow stimulation, which is similar to the process of pathological injury of human liver [15]. Additionally, unlike the New Zealand rabbit model, the Mongolian gerbil model does not experience slow self-healing in the stage of advanced liver fibrosis [16]. It is the most realistic pathological reproduction of patients with infectious liver fibrosis. Compared with the liver fibrosis mice model, individual dynamics of biochemical indicators in Mongolian gerbil can be more easily tracked and individual differences between the models could be maximally homogenized. At the same time, it greatly facilitates consistent analysis of liver fibrosis pathological data.

We evaluated the effect of ART against liver fibrosis in HSCs (LX-2 cells) in vitro and schistosomiasis Mongolian gerbil model in vivo. Subsequently, we screened differentially expressed genes (DEGs) related to ART treatment by whole transcriptomics sequencing (RNA-Seq) of liver tissues to explore the potential regulatory mechanisms of ART against schistosomiasis liver fibrosis.

Methods

Ethical Approval of the Study Protocol

Animal experiments were approved by the Laboratory Animal Welfare and Ethics Committee of Zhejiang Laboratory Animal Center (Zhejiang, China).

HSCs Proliferation and Apoptosis Detection

The antiproliferative effect of ART on HSCs was measured by CCK-8 assay. Briefly, HSCs (2000 cells per well) were seeded in 96-well plate and exposed to five different concentrations of ART (10, 20, 40, 60, 80, 100 and 120 μM), and the control and blank group were treated with equal volume PBS. After incubation for 24 h, each well was supplemented with 10 μL CCK-8 solution and incubated for 1 h. Cell viability was determined by detecting the absorbance at 450nm. Each experiment was performed in triplicate.

Apoptosis in HSCs was assessed by FITC/PI staining. Briefly, HSCs ($1x10^4$ cells per well) were seeded in 24-well plate and exposed to different concentrations of ART (40 and 80 μM) for 48 h. Cells were then added with the mixture of 195 μL Annexin V-FITC binding solution and 5 μL Annexin V-FITC, followed by addition of 10 μL PI pigment. After incubation for 10-20 min in the dark at 20-25°C, the cells were observed in the fluorescence microscope.

Animal Model and Artesunate Treatment

Forty clean grade male Mongolian gerbils (± 80g) were used in the experiment. Cercariae were induced and collected by exposing infected water-immersed snails with Schistosomiasis to light for 1.5 h and counted by direct observation under a light microscope. To established the animal model of schistosomiasis, 30 Mongolian gerbils were each infected with 80 *S. japonicum* cercariae by the abdominal patch method. Eight weeks post-infection, Mongolian gerbils infected with cercariae were administered by oral gavage with praziquantel diluted in 1% CMC-Na solution at the dose of 75 $mg/kg \cdot d^{-1}$ for 3 days. Twelve weeks post-infections, 30 Mongolian gerbils infected were randomly divided into 3 groups, model infection group (1% CMC-Na, MOD), low-dose ART treatment group (20$mg/kg \cdot d^{-1}$, ART-L) and high-dose ART treatment group (40$mg/kg \cdot d^{-1}$, ART-H). Mongolian gerbils in ART-L and ART-H group were treated by oral gavage with 0.2 mL ART solution with corresponding concentration; Mongolian gerbils in normal control (CON) and MOD group were administrated by oral gavage with 0.2 mL 1% CMC-Na solution, once daily for 12 weeks.

Histological Assessment and Biochemical Indicators Detection

Inflammation and fibrosis of liver were accessed by histological analysis and biochemical indicators of HA, ALT, malondialdehyde (MDA) and glutathione peroxidase (GPX) detection. Superior segment of left lateral lobe of each Mongolian gerbil liver was fixed in 10% formalin, embedded in paraffin, sectioned and stained with hematoxylin-eosin (H&E) and Masson's trichrome for light microscopy. The degree of inflammation and fibrosis was graded according to the Blueprint for the Diagnosis and Assessment of Therapeutic Efficacy in Liver Fibrosis Due to

Schistosomiasis, and Consensus on Evaluation of the Diagnosis and Efficacy of Hepatic Fibrosis in 2019. Serum HA/ALT levels were detected by using automatic biochemical analyzer to evaluate liver injury and liver function. Levels of MDA and activity of GPX in liver tissue were detected by spectrophotometer.

Whole Transcriptomics Sequencing

Total RNA was isolated ~~form~~ liver tissue by Trizol regent (Invitrogen, CA, USA). Then RNA concentration and purity were measured by using Nano drop 2000. RNA integrity was identified by agarose gel electrophoresis and Bioanalyzer 2100 (Agilent, CA, USA) and considered to be good with RIN >7.0. Small RNA library was constructed according to protocol of TruSeq Small RNA Sample Prep Kits (Illumina, San Diego, USA) and single-end sequencing was performed on the Illumina Hi-seq 2500. For strand-specific library construction and paired-end sequencing on the Illumina Hi-seq 4000, RNA samples were purified according to the manuscript of the Epicentre Ribo-Zero Gold Kit (Illumina, San Diego, USA) and fragmented into small pieces. The cleaved RNA fragments were then reverse-transcribed to create the final cDNA library in accordance with the protocol for the RNA-Seq sample preparation kit (Illumina, San Diego, USA).

Data Processing and Bioinformatics Analysis

Raw reads were processed by Cutadapt to genenrate clean reads and verified through FastQC (http://www.bioinformatics.babraham.ac.uk/projects/fastqc/). Clean reads were then mapped to the genome of mice and assembled using StringTie. Transcriptomes from Mongolian gerbil samples were merged to reconstruct a comprehensive transcriptome using perl scripts. The transcript-level expression level of RNA-seq was analyzed by calculating FPKM. DEGs were selected with log2 (fold change) >1 or log2 (fold change) < -1 and with statistical significance (p value < 0.05) by R package Ballgown. GO terms and KEGG pathways of these DEGs were annotated. Protein-protein interaction (PPI) network of DEGs was performed by the STRING online analysis tool (https://string-db.org/), PPI network was visualized and analyzed by Cytoscape software 3.7.2, and the TOP30 DEGs were selected as Hub genes. For miRNAs analysis, unique sequence with length in 18~26 nucleotide was mapped to specific species precursors in miRBase 21.0 by BLAST search to identify known miRNAs and novel 3p- and 5p- derived miRNAs. Target genes were predicted by TargetScan 8.0 and Miranda 3.3a.

Real-time Quantitative PCR

To validate RNA-Seq results, total RNA was extracted from liver tissue using Trizol regent. Reverse transcription for each sample was performed on 200 ng of total RNA by using cDNA reverse transcription kit (Takara, Beijing, China).

Thereafter, real-time fluorescent quantitative PCR was utilized to detect the expression levels of the candidate genes. The primers were designed with Primer 5.0 software (**S1 Table**) and synthesized by Tsingke Biotechnology Co., Ltd. Results were calculated by the $2^{-\Delta\Delta Ct}$ method and GAPDH were used as an internal reference gene for each example.

Statistics Analysis

Data were presented as the mean ± SEM. Statistical analyses were performed using SPSS 26.0 and Graph Pad Prism 8.4 software. Statistical comparison between groups was performed using one-way analysis of variance (ANOVA) with a Tukey test and student's t test. $P<0.05$ was considered statistically significant.

Results

Effect of Artesunate on HSCs and Animal Model

The effect of ART on HSCs was investigated by CCK-8 and FITC/PI double staining assay. Compared with control group, apoptotic morphological changes were observed after ART treatment under the microscope. Results showed that ART (10 μM) significantly inhibited HSCs viability in proliferation at 24 h after treatment ($p<0.05$ vs. control) (**Fig 1A and B**). With the concentration of ART increased, an increasing number of apoptosis cells were observed (**Fig 1C**). Figure 2A illustrates the respective timeline of the in vivo studies. In vivo, serum HA and ALT level were detected respectively after 8 weeks of infection and 12 weeks of ART treatment. In the ART-H group, HA and ALT serum level decreased after ART treatment although the differences were not statistically significant. Additionally, the GPX activity were significantly higher in the ART-H group than that of in the MOD group (**Fig 2B**). In appearance, the livers in the MOD group were chestnut black and had slightly increased volume, blunt edges. There was a marked improvement in liver and spleen morphology and color in the ART-H group whereas changes to liver and spleen index were not significant (data were not shown). Histopathological changes in livers were ~~accessed~~ by H&E and Masson staining. Compared with CON group, inflammatory cell infiltration and collagen fiber deposition were observed in the MOD group, while collagen deposition decreased significantly in the ART-H group, indicating that high-dose of ART administration have protective effect against liver fibrosis (**Fig 2C**).

Fig 1. The effect of Artesunate on HSCs. (A) HSCs morphology after 24 h of ART intervention with different concentrations under an inverted phase-contrast microscope (100X); (B) Viability of HSCs incubated with different concentrations of ART for 24 h; (C) Fluorescence microscopy images of apoptosis cell after 24 h of ART treatment with different concentration of ART (a: 40 μM and b: 80 μM), 100X. Data are presented as mean ± SEM (n=3); ***$p<0.001$ (ART treatment vs control

group).

Fig 2. The effect of Artesunate on Schistosoma-induced liver fibrosis in Mongolian gerbil. (A) Experimental timeline of in vivo; (B) Levels of HA, ALT, GPX and MDA; (C) Liver appearance and pathological changes of liver tissue (200X), HE staining and Masson staining score statistics. Data were presented as mean ± SEM (n=4 per group). *p<0.05 (after administration vs before administration).

Transcriptome Expression Profiles of Schistosomiasis Liver Fibrosis in Response to Artesunate

For each sequencing library, the reads of Q20, Q30 and GC content exceeded 99%, 96% and 47% respectively, which indicated a high quality of clean data (**S2 Table**). At the same time, more than 90% of the total reads were successfully mapped to the reference genome, of which more than 64% of readings are uniquely located (**S3 Table**). Then uniquely mapped reads were used for subsequent analysis. DEGs were then screened for subsequent analysis. Compared with CON group, 467 genes were up-regulated and 144 genes were down-regulated in the MOD group, 187 genes were up-regulated and 142 genes were down-regulated in the ART group. Compared ART group with MOD group，117 genes were up-regulated and 216 genes were down-regulated (**Fig 3A**). The shared and unique DEGs between different groups were ~~investigated~~ by Venn diagram. DEGs between MOD vs CON and ART vs MOD, except for ART vs CON, were considered ART treatment-related genes, which indicated 158 DEGs related to ART effect against schistosomiasis liver fibrosis (**Fig 3B**). The heat map reveled differential expression pattern between different group (**Fig 3C**). And we also identified 131 differentially expressed miRNAs related to ART anti-schistosomiasis liver fibrosis **(S1 Fig. and S4 Table)**. The expression trend of qPCR targeted Ctss, Mgp, Vim, Ctgf, Cd74, Chi3l1 was consistent with the sequencing results, which indicating that the sequencing results are reliable (**Fig 3D**).

Fig 3. Differentially expressed genes screening and validation. (A) Volcano plot, (B) Venn diagram and (C) Heat map of differentially expressed genes (ART_1: ART-L group; ART_2: ART-H group); (D) qPCR Validation of differentially expressed genes. Data were presented as mean ± SEM (n=2); ###p<0.001 (MOD group vs CON group); ***p<0.001 (ART group vs MOD group).

Enrichment Analysis of DEGs and PPI Analysis

Subsequently, 158 DEGs were conducted respectively by GO and KEGG enrichment analysis to explore the potential mechanism of ART. The top 20 significant GO and pathway terms are displayed (**Fig 4A and B**). GO analysis demonstrated that these DEGs were mainly enriched in immune response, inflammatory response and integrin-mediated signaling pathway et al (**Fig 4A**).

KEGG pathway analysis of these DEGs were mainly enriched in complement and coagulation cascades, chemokine signaling pathway, Fc gamma R-mediated phagocytosis, focal adhesion, platelet activation, toll-like receptor signaling pathway and HIF-1 signaling pathway (**Fig 4B**). PPI network of 158 DEGs generated 109 nodes and 271 edges (p<1.0-16), and TOP 30 DEGs were selected as Hub genes (**Fig 4C**). Most of the predicted target genes of 6 miRNAs (miR-193a-3p, miR-370-3p, miR-365-3p, miR-2137, miR-3473b, miR-3473e) are also differentially expressed Hub genes related to ART anti-schistosomiasis liver fibrosis, including Tlr4, Cyth4, Irf8, Nckap1l, Myo1F, Src and Fscn1, Adamtsll2, Prkcd, Surf4 and Ddit4 (https://www.targetscan.org/vert_80/). These DEGs implicate central pathways, such as immune and inflammatory response, integrin-mediated signaling pathway and toll-like receptor signaling pathway~~, et al~~.

Fig 4. GO and KEGG enrichment and PPI analysis of differentially expressed genes. (A) GO enrichment analysis; (B) KEGG enrichment analysis; (C) (left) protein-protein interaction analysis, blue: down-regulation, red: up-regulation; (right) Hub gene screening, interactions degree_top30 as Hub genes, dark red represents a high degree of connectivity.

Fig 5. Potential candidate regulators of Schistosomiasis liver fibrosis in response to Artesunate therapy. The red arrows indicate upregulated genes and the green arrows indicate downregulated genes; asterisk indicates genes reported previously.

Discussion

ART has achieved more attention because of its various pharmacological properties. Studies have shown that ART has significant anti-fibrosis activity, while the specific molecular mechanism has not been systematically elucidated [17]. We investigated the effect of ART on liver fibrosis in HSCs and infectious Mongolian gerbil model. Mechanistically, RNA-Seq analysis of liver tissue was performed to explore the molecular mechanism of ART on liver fibrosis.

ART can significantly inhibit the proliferation and induce apoptosis of HSCs in vitro and alleviate effectively schistosomiasis liver fibrosis in vivo in this study. In HSCs, ART significantly inhibited cell proliferation in a dose- dependent manner within a concentration range from 10μM to 120μM. In addition, the number of apoptotic cells gradually increased with the increased concentration of ART, indicating that ART promotes their apoptosis in a dose-dependent manner. The latest research also showed that ART promotes HSCs apoptosis by inhibiting mitochondrial function [14]. In order to explore the anti-fibrosis activity of ART in vivo, we successfully constructed the liver fibrosis experimental model in Mongolian gerbil infected with *S. japonicum*. The liver index were improved slightly by ATR gavage treatment, and pathological changes of liver tissue showed that inflammation cells infiltration and collage deposition were reduced, and similar findings have been reported from one previous study suggesting a decreased area of fibrosis and granuloma in ART intervention group in the mouse model [14], which indicating that ART relieves schistosomiasis liver fibrosis to some extent.

To further explore the anti-fibrosis mechanism of ART, we performed

transcriptome RNA-Seq of liver from CON, MOD and ART group. Preliminary cluster analysis revealed slight individual gene expression differences in MOD group, which may be caused by the fact that the number of infected schistosome cercariae was not strictly controlled. Further bioinformatics analysis determined 158 DEGs related to ART anti-schistosomiasis liver fibrosis. GO function analysis showed that these DEGs were involved in immune response, extracellular space and calcium ion transportation terms. KEGG pathway analysis revealed that immune response, focal adhesion, platelet activation and HIF-1 signaling pathway were significantly enriched.

Current strategies of treatment of liver fibrosis are directly mainly to inhibit HSC activation, induce activated HSC apoptosis, increase ECM degradation, and suppress inflammation (**Fig 5**). Activation of HSCs is considered to be a key event in liver fibrosis and TGF-β1 is responsible for HSCs activation. Src family tyrosine kinases are activated in different organs fibrosis such as lung, kidney and liver, and considered to be the amplifiers of TGF-β1 signal in the pathological process of fibrosis [18]. The activated Src under hypoxic conditions of tissue cells can mediate the expression of Ctgf induced by TGF-β1, further stimulate collagen secretion and eventually accelerate fibrosis process [19, 20]. At present, Src has become a potential therapeutic target for various organ fibrosis. For example, Sarakatinib, a tyrosine kinase activity inhibitor, has been approved by the FDA for the treatment of idiopathic pulmonary fibrosis[21, 22]. Our RNA-seq results showed that Src is significantly down regulated in ART group compared to MOD group, which indicted that Src is a potential target of ART to liver fibrosis [23](Berkoz et al. 2021). Additionally, previous research reported that ART prevent schistosomiasis liver fibrosis by regulating mitochondrial function in HSC [7, 14]. We identified 6 mitochondrial-related differentially expressed genes (Ddit4, Ndufa7, Higd2a, Ucp2, Mrpl36, Fahd1) in this study, which are potential targets of ART. The DNA damage-induced transcript Ddit4 is expressed under stress and is located downstream of HIF-1α, which negatively regulates mTOR-induced cell proliferation and induces autophagy [24, 25]. Ctss is one of the TOP30 DEGs in this study and it promotes cell migration and invasion by regulating Ca^{2+} homeostasis and ECM degradation [26]. Studies have shown that inhibiting Ctss could increase the expression of mitochondrial calcium uniporter and enhance the absorption capacity of mitochondrial Ca^{2+}, leading to mitochondrial Ca^{2+} overload, the production of large amounts of ROS, and finally cell apoptosis mediated by mitochondria [27]. However, further experimental verification in vivo is required to confirm this interesting hypothesis.

Excessive deposition of ECM is the main pathological feature of liver fibrosis, and thus increasing ECM degradation is a pivotal strategy for antifibrotic therapies. In our RNA-Seq and PPI analysis data, several DEGs participate in the regulation of actin cytoskeleton assembly and ECM adhesion, such as Ctgf, Qsox1, Fscn1, Myo1f, Lpxn, Scin, Parvg, Itgb2 and Fermt3 [28-31]. Interestingly, Ctgf has been reported upregulated in various fibrosis diseases including lung, kidney, skin and liver fibrosis [32, 33]. Inhibition of Ctgf inactivated HSCs and reduced ECM formation [34, 35].

Therefore, Ctgf may be a potential intervention target of liver fibrosis reverse.

Conclusion

We developed liver fibrosis model in Mongolian gerbil of mimicking human infection with S. japonicum cercariae. The efficacy test showed that ART is effective in preventing liver fibrosis in vitro and in vivo. ART may alleviate effectively schistosomiasis liver fibrosis by decrease inflammation and collagen fibers deposition. We established the first comprehensive transcription profile, mined host candidate regulators and provided an overview of the complex network of the mechanism of Schistosomiasis liver fibrosis in response to ART. It is essential for understanding the effective mechanism of ART and for verifying their potential as diagnostic markers or novel therapeutic agents for Schistosomiasis liver fibrosis.

Conflict of interest

The authors declare that the research was conducted in the absence of any commercial or financial relationships that could be construed as a potential conflict of interest.

Author contributions

YY wrote the initial draft of the paper and analyzed the data. XL collected and analyzed the data. FD provided animals, experimental facilities and laboratory space. YY, HD, BZ, WZ, QT, JD, DL, XC and RC performed the experiments. QK designed the study, reviewed and edited the paper. QK, SL administrated the project and funding. All authors contributed to the article and approved the submitted version.

Funding

This research was supported by National Natural Science Foundation of China (31501050), Zhejiang Provincial Program for the Cultivation of High-level Innovative Health Talents (WJW2021002), Science and technology project of Zhejiang Province (GF22H188038) and the Zhejiang Provincial Natural Science Foundation of China (LQY19H190002).

Supplementary Information and Data Availability

S1-S4 Tables and S1 Fig. can be found in the Supporting Information files. Raw sequencing data of RNA were uploaded on Sequencing Read Archive of NCBI (Project number PRJNA846574).

Reference

1. Friedman SL. Hepatic fibrosis -- overview. Toxicology. 2008;254(3):120-9. Epub 2008/07/30.

doi: 10.1016/j.tox.2008.06.013. PubMed PMID: 18662740.

2. Lee YA, Wallace MC, Friedman SL. Pathobiology of liver fibrosis: a translational success story. Gut. 2015;64(5):830-41. Epub 2015/02/15. doi: 10.1136/gutjnl-2014-306842. PubMed PMID: 25681399; PubMed Central PMCID: PMCPMC4477794.

3. Chen RJ, Wu HH, Wang YJ. Strategies to prevent and reverse liver fibrosis in humans and laboratory animals. Arch Toxicol. 2015;89(10):1727-50. Epub 2015/05/13. doi: 10.1007/s00204-015-1525-6. PubMed PMID: 25963329.

4. Mahdinloo S, Kiaie SH, Amiri A, Hemmati S, Valizadeh H, Zakeri-Milani P. Efficient drug and gene delivery to liver fibrosis: rationale, recent advances, and perspectives. Acta Pharm Sin B. 2020;10(7):1279-93. Epub 2020/09/03. doi: 10.1016/j.apsb.2020.03.007. PubMed PMID: 32874828; PubMed Central PMCID: PMCPMC7451940.

5. Chen J, Hu Y, Chen L, Liu W, Mu Y, Liu P. The effect and mechanisms of Fuzheng Huayu formula against chronic liver diseases. Biomed Pharmacother. 2019;114:108846. Epub 2019/04/10. doi: 10.1016/j.biopha.2019.108846. PubMed PMID: 30965233.

6. Tian H, Liu L, Li Z, Liu W, Sun Z, Xu Y, et al. Chinese medicine CGA formula ameliorates liver fibrosis induced by carbon tetrachloride involving inhibition of hepatic apoptosis in rats. J Ethnopharmacol. 2019;232:227-35. Epub 2018/11/25. doi: 10.1016/j.jep.2018.11.027. PubMed PMID: 30471378.

7. Luo JT, Qian SN, Wu KY, Zhu SC, Huang YL, Ye JP, et al. [Exploring the effects of artesunate and fuzheng huayu decoction on mitochondria in the treatment of schistosomiasis liver fibrosis]. Zhonghua Gan Zang Bing Za Zhi. 2022;30(1):45-51. Epub 2021/02/26. doi: 10.3760/cma.j.cn501113-20201024-00577. PubMed PMID: 33626860.

8. Vatsveen TK, Myhre MR, Steen CB, Walchli S, Lingjaerde OC, Bai B, et al. Artesunate shows potent anti-tumor activity in B-cell lymphoma. J Hematol Oncol. 2018;11(1):23. Epub 2018/02/21. doi: 10.1186/s13045-018-0561-0. PubMed PMID: 29458389; PubMed Central PMCID: PMCPMC5819282.

9. Yan G, Dawood M, Bockers M, Klauck SM, Fottner C, Weber MM, et al. Multiple modes of cell death in neuroendocrine tumors induced by artesunate. Phytomedicine. 2020;79:153332. Epub 2020/09/22. doi: 10.1016/j.phymed.2020.153332. PubMed PMID: 32957040.

10. Zeng XZ, Zhang YY, Yang Q, Wang S, Zou BH, Tan YH, et al. Artesunate attenuates LPS-induced osteoclastogenesis by suppressing TLR4/TRAF6 and PLCgamma1-Ca(2+)-NFATc1 signaling pathway. Acta Pharmacol Sin. 2020;41(2):229-36. Epub 2019/08/23. doi: 10.1038/s41401-019-0289-6. PubMed PMID: 31431733; PubMed Central PMCID: PMCPMC7468527.

11. Lai L, Chen Y, Tian X, Li X, Zhang X, Lei J, et al. Artesunate alleviates hepatic fibrosis induced by multiple pathogenic factors and inflammation through the inhibition of LPS/TLR4/NF-kappaB signaling pathway in rats. Eur J Pharmacol. 2015;765:234-41. Epub 2015/09/01. doi: 10.1016/j.ejphar.2015.08.040. PubMed PMID: 26318197.

12. Lv J, Bai R, Wang L, Gao J, Zhang H. Artesunate may inhibit liver fibrosis via the FAK/Akt/beta-catenin pathway in LX-2 cells. BMC Pharmacol Toxicol. 2018;19(1):64. Epub 2018/10/18. doi: 10.1186/s40360-018-0255-9. PubMed PMID: 30326962; PubMed Central PMCID: PMCPMC6192352.

13. Kong Z, Liu R, Cheng Y. Artesunate alleviates liver fibrosis by regulating ferroptosis signaling pathway. Biomed Pharmacother. 2019;109:2043-53. Epub 2018/12/16. doi:

10.1016/j.biopha.2018.11.030. PubMed PMID: 30551460.

14. Shen S, Luo J, Ye J. Artesunate alleviates schistosomiasis-induced liver fibrosis by downregulation of mitochondrial complex subunit NDUFB8 and complex subunit UQCRC2 in hepatic stellate cells. Acta Trop. 2021;214:105781. Epub 2020/12/03. doi: 10.1016/j.actatropica.2020.105781. PubMed PMID: 33264632.

15. Liang YR, Guo YX, Yi XY, Zeng XF, Peng LX, Zeng QS. Schistosoma japonicum: a comparison of the development of the parasite and associated pathological changes in mice and jirds (Meriones unguiculatus). Int J Parasitol. 1983;13(6):531-8. Epub 1983/12/01. doi: 10.1016/s0020-7519(83)80024-1. PubMed PMID: 6654579.

16. Dunn MA, Cheever AW, Paglia LM, Kelly EP, Duvall RH, Andrade ZA, et al. Reversal of advanced liver fibrosis in rabbits with Schistosomiasis japonica. Am J Trop Med Hyg. 1994;50(4):499-505. Epub 1994/04/01. doi: 10.4269/ajtmh.1994.50.499. PubMed PMID: 8166357.

17. Wang Y, Wang Y, You F, Xue J. Novel use for old drugs: The emerging role of artemisinin and its derivatives in fibrosis. Pharmacol Res. 2020;157:104829. Epub 2020/05/04. doi: 10.1016/j.phrs.2020.104829. PubMed PMID: 32360483.

18. Wang J, Zhuang S. Src family kinases in chronic kidney disease. Am J Physiol Renal Physiol. 2017;313(3):F721-F8. Epub 2017/06/16. doi: 10.1152/ajprenal.00141.2017. PubMed PMID: 28615246; PubMed Central PMCID: PMCPMC5625110.

19. Mishra R, Zhu L, Eckert RL, Simonson MS. TGF-beta-regulated collagen type I accumulation: role of Src-based signals. Am J Physiol Cell Physiol. 2007;292(4):C1361-9. Epub 2006/12/01. doi: 10.1152/ajpcell.00370.2006. PubMed PMID: 17135298.

20. Cheng Z, Liu L, Wang Z, Cai Y, Xu Q, Chen P. Hypoxia Activates Src and Promotes Endocytosis Which Decreases MMP-2 Activity and Aggravates Renal Interstitial Fibrosis. Int J Mol Sci. 2018;19(2). Epub 2018/02/22. doi: 10.3390/ijms19020581. PubMed PMID: 29462885; PubMed Central PMCID: PMCPMC5855803.

21. Li LF, Kao KC, Liu YY, Lin CW, Chen NH, Lee CS, et al. Nintedanib reduces ventilation-augmented bleomycin-induced epithelial-mesenchymal transition and lung fibrosis through suppression of the Src pathway. J Cell Mol Med. 2017;21(11):2937-49. Epub 2017/06/10. doi: 10.1111/jcmm.13206. PubMed PMID: 28598023; PubMed Central PMCID: PMCPMC5661114.

22. Liu F, Wang L, Qi H, Wang J, Wang Y, Jiang W, et al. Nintedanib, a triple tyrosine kinase inhibitor, attenuates renal fibrosis in chronic kidney disease. Clin Sci (Lond). 2017;131(16):2125-43. Epub 2017/06/25. doi: 10.1042/CS20170134. PubMed PMID: 28646122.

23. Berkoz M, Ozkan-Yilmaz F, Ozluer-Hunt A, Krosniak M, Turkmen O, Korkmaz D, et al. Artesunate inhibits melanoma progression in vitro via suppressing STAT3 signaling pathway. Pharmacol Rep. 2021;73(2):650-63. Epub 2021/02/21. doi: 10.1007/s43440-021-00230-6. PubMed PMID: 33609273.

24. Chen R, Wang B, Chen L, Cai D, Li B, Chen C, et al. DNA damage-inducible transcript 4 (DDIT4) mediates methamphetamine-induced autophagy and apoptosis through mTOR signaling pathway in cardiomyocytes. Toxicol Appl Pharmacol. 2016;295:1-11. Epub 2016/01/31. doi: 10.1016/j.taap.2016.01.017. PubMed PMID: 26825372.

25. Gharibi B, Ghuman M, Hughes FJ. DDIT4 regulates mesenchymal stem cell fate by mediating between HIF1alpha and mTOR signalling. Sci Rep. 2016;6:36889. Epub 2016/11/24. doi: 10.1038/srep36889. PubMed PMID: 27876894; PubMed Central PMCID: PMCPMC5120275.

26. Lin HH, Chen SJ, Shen MR, Huang YT, Hsieh HP, Lin SY, et al. Lysosomal cysteine protease

cathepsin S is involved in cancer cell motility by regulating store-operated Ca(2+) entry. Biochim Biophys Acta Mol Cell Res. 2019;1866(12):118517. Epub 2019/07/25. doi: 10.1016/j.bbamcr.2019.07.012. PubMed PMID: 31340164.

27. Fei M, Zhang L, Wang H, Zhu Y, Niu W, Tang T, et al. Inhibition of Cathepsin S Induces Mitochondrial Apoptosis in Glioblastoma Cell Lines Through Mitochondrial Stress and Autophagosome Accumulation. Front Oncol. 2020;10:516746. Epub 2021/01/12. doi: 10.3389/fonc.2020.516746. PubMed PMID: 33425712; PubMed Central PMCID: PMCPMC7787074.

28. Gupta A, Lee BS, Khadeer MA, Tang Z, Chellaiah M, Abu-Amer Y, et al. Leupaxin is a critical adaptor protein in the adhesion zone of the osteoclast. J Bone Miner Res. 2003;18(4):669-85. Epub 2003/04/04. doi: 10.1359/jbmr.2003.18.4.669. PubMed PMID: 12674328.

29. Yoshimi R, Yamaji S, Suzuki A, Mishima W, Okamura M, Obana T, et al. The gamma-parvin-integrin-linked kinase complex is critically involved in leukocyte-substrate interaction. J Immunol. 2006;176(6):3611-24. Epub 2006/03/07. doi: 10.4049/jimmunol.176.6.3611. PubMed PMID: 16517730.

30. Navines-Ferrer A, Martin M. Long-Tailed Unconventional Class I Myosins in Health and Disease. Int J Mol Sci. 2020;21(7). Epub 2020/04/11. doi: 10.3390/ijms21072555. PubMed PMID: 32272642; PubMed Central PMCID: PMCPMC7177449.

31. Ou C, Sun Z, He X, Li X, Fan S, Zheng X, et al. Targeting YAP1/LINC00152/FSCN1 Signaling Axis Prevents the Progression of Colorectal Cancer. Adv Sci (Weinh). 2020;7(3):1901380. Epub 2020/02/12. doi: 10.1002/advs.201901380. PubMed PMID: 32042551; PubMed Central PMCID: PMCPMC7001651.

32. Gressner OA, Gressner AM. Connective tissue growth factor: a fibrogenic master switch in fibrotic liver diseases. Liver Int. 2008;28(8):1065-79. Epub 2008/09/12. doi: 10.1111/j.1478-3231.2008.01826.x. PubMed PMID: 18783549.

33. Shi C, Li G, Tong Y, Deng Y, Fan J. Role of CTGF gene promoter methylation in the development of hepatic fibrosis. Am J Transl Res. 2016;8(1):125-32. Epub 2016/04/14. PubMed PMID: 27069546; PubMed Central PMCID: PMCPMC4759422.

34. Yuhua Z, Wanhua R, Chenggang S, Jun S, Yanjun W, Chunqing Z. Disruption of connective tissue growth factor by short hairpin RNA inhibits collagen synthesis and extracellular matrix secretion in hepatic stellate cells. Liver Int. 2008;28(5):632-9. Epub 2008/04/25. doi: 10.1111/j.1478-3231.2008.01730.x. PubMed PMID: 18433392.

35. Hao C, Xie Y, Peng M, Ma L, Zhou Y, Zhang Y, et al. Inhibition of connective tissue growth factor suppresses hepatic stellate cell activation in vitro and prevents liver fibrosis in vivo. Clin Exp Med. 2014;14(2):141-50. Epub 2013/03/05. doi: 10.1007/s10238-013-0229-6. PubMed PMID: 23456538.

Supporting information

S1 Fig. Differentially expressed miRNAs. (A) Heat map and (B) Venn diagram of differentially expressed genes; ART_1: ART-L group, ART_2: ART-H group.

S1 Table. Primer information.

S2 Table. Quality parameters of clean data.

S3 Table. Number of unique mapped reads.

S4 Table. Differentially expressed miRNAs.
