# Supplementary material for: Mining host candidate regulators of schistosomiasis-induced liver fibrosis in response to artesunate therapy through transcriptomics approach
Source: PLoS Negl Trop Dis. 2023 Sep 29;17(9):e0011626. doi: 10.1371/journal.pntd.0011626 (PMC10566724; doi:10.1371/journal.pntd.0011626)
Supplement: Attachment — Submitted filename: PNTD-D-22-01236_reviewer.pdf [file pntd.0011626.s009.pdf]

PLOS Neglected Tropical Diseases

Mining Host Candidate Regulators of Schistosomiasis-Induced Liver Fibrosis in Response to Artesunate Therapy through Transcriptomics Approach

--Manuscript Draft--

**Manuscript Number:**	PNTD-D-22-01236R1
**Full Title:**	Mining Host Candidate Regulators of Schistosomiasis-Induced Liver Fibrosis in Response to Artesunate Therapy through Transcriptomics Approach
**Short Title:**	ART regulates schistosomiasis-induced liver fibrosis.
**Article Type:**	Research Article
**Keywords:**	Schistosoma; infectious animal model; liver fibrosis; hepatic stellate cells; artesunate
**Abstract:**	Background: Artesunate (ART) has been reported to have an antifibrotic effect in various organs. The underlying mechanism has not been systematically elucidated. We aimed to clarify the effect of ART on liver fibrosis induced by Schistosoma japonicum (S.japonicum) in an experimentally infected rodent model and the potential underlying mechanisms. Methods: The effect of ART on hepatic stellate cells (HSCs) was assessed using CCK-8 and Annexin V-FITC/PI staining assays. The experimental model of liver fibrosis was established in the Mongolian gerbil model infected with S.japonicum cercariae and then treated with 20 mg/kg or 40 mg/kg ART. The hydroxyproline (Hyp) content, malondialdehyde (MDA) content, superoxide dismutase (SOD) and glutathione peroxidase (GPX) activities in liver tissue were measured and histopathological changes of liver tissues were observed. Whole-transcriptome RNA sequencing (RNA-seq)of the liver tissues was performed. Differentially expressed genes (DEGs) were identified using bioinformatic analysis and verified by quantitative PCR (qPCR) and western blot assay. Results: ART significantly inhibited the proliferation and induce the apoptosis of HSCs in a dose-dependent manner. In vivo, Hyp content decreased significantly in the ART-H group compared to the MOD group and GPX activity was significantly higher in the ART-H group than in the MOD group. Besides, ART treatment significantly reduced collagen production ($p < 0.05$). A total of 158 DEGs and 44 differentially expressed miRNAs related to ART-induced anti-schistosomiasis liver fibrosis were identified. The qPCR and western blot results of selected DEGs were consistent with the sequencing results. These DEGs were implicated in key pathways such as immune and inflammatory response, integrin-mediated signaling and toll-like receptor signaling pathways. Conclusion: ART is effective against liver fibrosis using Mongolian gerbil model induced by S. japonicum infection. We identified host candidate regulators of schistosomiasis-induced liver fibrosis in response to ART through transcriptomics approach.
**Additional Information:**	
**Question**	**Response**
**Government Employee**<br><br>Are you or any of the contributing authors an employee of the United States government?<br><br>Manuscripts authored by one or more US Government employees are not copyrighted, but are licensed under a CC0 Public Domain Dedication, which allows unlimited distribution and reuse of the article for any lawful purpose. This is a legal requirement for US Government	No - No authors are employees of the U.S. government.

Powered by Editorial Manager® and ProduXion Manager® from Aries Systems Corporation

employees.<br><br>This will be typeset if the manuscript is accepted for publication.	
**Financial Disclosure**<br><br>Enter a financial disclosure statement that describes the sources of funding for the work included in this submission and the role the funder(s) played. This includes grants and any commercial funding of the work or authors.<br><br>This statement will be typeset if the manuscript is accepted for publication.<br><br>Review the submission guidelines and the instructions link below for detailed requirements and guidance.	This study was supported by the National Natural Science Foundation of China (QK., NO. 31501050); Zhejiang Provincial Program for the Cultivation of High-Level Innovative Health Talents (QK., NO. WJW2021002); Basic Public Welfare Research Project of Zhejiang Province (FD., NO. LGF22H180015); Medical Health Science and Technology Project of Zhejiang Provincial Health Commission (FD., NO. 2020KY530); Zhejiang Provincial Natural Science Foundation of China (HD., NO. LQY19H190002). The funders had no role in study design, data collection and analysis, decision to publish, or preparation of the manuscript. No author received a salary from any of the funders.
**Competing Interests**<br><br>On behalf of all authors, disclose any competing interests that could be perceived to bias this work.<br><br>This statement will be typeset if the manuscript is accepted for publication.<br><br>Review the instructions link below and PLOS NTDs' competing interests policy to determine what information must be disclosed at submission.	The authors have declared that no competing interests exist.
**Data Availability**<br><br>Provide a **Data Availability Statement** in the box below. This statement should detail where the data used in this submission can be accessed. This statement will be typeset if the manuscript is accepted for publication.<br><br>Before publication, authors are required to	The RNA-Seq raw data data that support the findings of this study are publicly available from [Sequencing Read Archive of NCBI] with the identifier(s) [Project number PRJNA846574] once the paper is published. All other relevant data are within the manuscript and its Supporting Information files.

make all data underlying their findings fully available, without restriction. Review our PLOS Data Policy page for detailed information on this policy. Instructions for writing your Data Availability statement can be accessed via the Instructions link below.	

Manuscript

Click here to access/download;Manuscript;Regulators of Schistosomiasis Liver Fibrosis to Artesunate.pdf

Mining Host Candidate Regulators of Schistosomiasis-Induced Liver Fibrosis in Response to Artesunate Therapy through Transcriptomics Approach

Yajie Yuan [1 †], Xinyue Lv [1 †], Fangwei Dai [2], Haojie Ding [3], Riping Chen [4], Bin Zheng [3], Wenxia Zhao [4], Qunbo Tong [3], Jianzu Ding [3], Di Lou [3], Xiaofeng Chu [2], Shaohong Lu [3 *] and Qingming Kong [1, 3, *]

[1] School of Laboratory Medicine and Bioengineering, Hangzhou Medical College, Zhejiang Academy of Medical Sciences, Hangzhou, China

[2] Zhejiang Key Laboratory of Experimental Animal and Safety Evaluation, Hangzhou Medical College, Zhejiang Academy of Medical Sciences, Hangzhou, China

[3] Institute of Parasitic Diseases, Hangzhou Medical College, Zhejiang Academy of Medical Sciences, Hangzhou, China

[4] School of Public Health, Hangzhou Medical College, Zhejiang Academy of Medical Sciences, Hangzhou, China

[†] These authors contributed equally to this work.

* **Correspondence:** Shaohong Lu, Email: llsshh2003@163.com; Qingming Kong, Email: qmkong_1025@163.com

Abstract

Background: Artesunate (ART) has been reported to have an antifibrotic effect in various organs. The underlying mechanism has not been systematically elucidated. We aimed to clarify the effect of ART on liver fibrosis induced by *Schistosoma japonicum* (*S. japonicum*) in an experimentally infected rodent model and the potential underlying mechanisms. **Methods:** The effect of ART on hepatic stellate cells (HSCs) was assessed using CCK-8 and Annexin V-FITC/PI staining assays. The experimental model of liver fibrosis was established in the Mongolian gerbil model infected with *S. japonicum* cercariae and then treated with 20 mg/kg or 40 mg/kg ART. The hydroxyproline (Hyp) content, malondialdehyde (MDA) content, superoxide dismutase (SOD) and glutathione peroxidase (GPX) activities in liver tissue were measured and histopathological changes of liver tissues were observed. Whole-transcriptome RNA sequencing (RNA-seq) of the liver tissues was performed. Differentially expressed genes (DEGs) were identified using bioinformatic analysis and verified by quantitative PCR (qPCR) and western blot assay. **Results:** ART significantly inhibited the proliferation and induce the apoptosis of HSCs in a dose-dependent manner. *In vivo*, Hyp content decreased significantly in the ART-H group compared to the MOD group and GPX activity was significantly higher in the ART-H group than in the M[illegible] group. Besides, ART treatment significantly reduced collagen production ($p < 0.05$). A total of 158 DEGs and 44 differentially expressed miRNAs related to ART-induced anti-schistosomiasis liver fibrosis were identified. The qPCR and western blot results of selected DEGs were consistent with the sequencing results. These DEGs were implicated in key pathways such as immune and inflammatory response, integrin-mediated signaling and toll-like receptor signaling pathways. **Conclusion:** ART is effective against liver fibrosis using Mongolian gerbil model induced by *S. japonicum* infection. We identified host candidate regulators of schistosomiasis-induced liver fibrosis in response to ART through transcriptomics approach.

Keywords: *Schistosoma*, infectious animal model, liver fibrosis, hepatic stellate cells, artesunate

Author summary

Artesunate (ART) has been reported to have an antifibrotic effect while the underlying mechanism has not been systematically elucidated. Here, we evaluated the effect of ART on anti-hepatic fibrosis in vitro and in vivo. We found that ART significantly inhibited the proliferation and induced the apoptosis of HSCs. And also ART is effective against liver fibrosis using Mongolian gerbil model induced by *Schistosoma japonicum* (*S. japonicum*) infection. Hyp content decreased significantly in the ART-H group compared to the MOD group and GPX activity was significantly higher in the ART-H group than in the MOD group. Besides, ART treatment

significantly reduced collagen production ($p<0.05$). A total of 158 DEGs and 44 differentially expressed miRNAs related to ART-induced anti-schistosomiasis liver fibrosis were identified through transcriptomics approach. These DEGs were implicated in key pathways such as immune and inflammatory response, integrin-mediated signaling and toll-like receptor signaling pathways. Our findings suggest that ART is effective against liver fibrosis. Furthermore, we identified 30 host candidate regulators in response to ART, and provides a basis for a better understanding of the molecular mechanisms of ART on anti-hepatic fibrosis.

Introduction

Liver fibrosis is a very complex dynamic pathological process characterized by the activation of hepatic stellate cells (HSCs), immunity and inflammation, increased extracellular matrix (ECM) proteins and fibrogenesis, which involves the activation or inhibition of multiple signaling pathways in various cell types [1]. ~~The~~ major risk factors for liver fibrosis include hepatitis virus infection, *Schistosoma japonicum* infection, alcoholic liver disease, drugs and metabolic disorders [2]. Numerous studies on the mechanism of liver fibrosis have focused mainly on HSCs activation, and thus activated HSCs have been considered to be an antifibrotic target [3]. Although several drugs or small molecules are in clinical trials, very few have been approved for the treatment of liver fibrosis [4].

Dozens of natural compounds exert antifibrotic effects in various liver fibrosis models *in vivo* and *in vitro*. Fuzheng Huayu (FZ[illegible]) can attenuate liver fibrosis by inhibiting HSCs activation and inflammatory response, regulating ECM deposition and inhibiting hepatic sinusoidal capillarization and angiogenesis, and thus it has been approved by China food and drug administration for treatment of liver fibrosis [5, 6]. Recently, Luo et al showed that FZHY and artesunate (ART) could alleviate schistosomiasis-induced liver fibrosis by modulating mitochondrial autophagy [7]. ART, a stable derivative of artemisinin extracted from the stems and leaves of *Artemisia annua*, has multiple pharmacological effects, including anti-inflammatory, antitumor and antifibrotic activities [8-10]. Additionally, ART can inhibit fibrosis through different mechanisms involving inflammatory infiltration, autophagy of ferritin, collagen synthesis, HSCs apoptosis, etc. [11-13]. Several studies have also proven that ART prevents schistosomiasis-induced liver fibrosis by regulating mitochondrial function [7, 14]. At present, existing studies on antifibrotic mechanisms of ART remain fragmented.

Mongolian gerbils are relatively suitable hosts of *S. japonicum*. The schistosomiasis-induced hepatic fibrosis in Mongolian gerbil is a very slow process because of continuous secreting soluble egg antigen (SEA) stimulation from eggs, which is similar to the process of pathological injury of the human liver [15]. Additionally, unlike the New Zealand rabbit model, the Mongolian gerbil model does not experience slow self-healing in the stage of advanced liver fibrosis [16]. It is the most realistic pathological reproduction of patients with infectious liver

fibrosis.

Herein, we evaluated the effect of ART on liver fibrosis in *in vitro* and *in vivo*. Subsequently, differentially expressed genes (DEGs) related to ART treatment were screened using whole transcriptome sequencing (RNA-seq) of liver tissues to explore potential regulatory mechanisms underlying the effect of ART on schistosomiasis-induced liver fibrosis.

Methods

Ethical Approval of the Study Protocol

Animal experiments were approved by the Laboratory Animal Welfare and Ethics Committee of Zhejiang Laboratory Animal Center (Zhejiang, China).

HSCs Proliferation and Apoptosis Detection

The LX-2 cell line generated by Xu et al. shows phenotypic features of activated HSCs [17]. Activated HSCs are the main source of the ECM, and proliferation inhibition or apoptosis of activated HSCs can reduce ECM production and thus alleviate fibrosis. The Cell Counting Kit-8 (CCK-8) assay was used to detect the proliferation of ART-treated HSCs. Briefly, HSCs (2000 cells/well) were seeded into 96-well plates and exposed to different concentrations of ART (10, 20, 40, 60, 80, 100 and 120 μM), and the control and blank groups were treated with an equal volume of phosphate-buffered saline. After incubation for 24 h, each well was supplemented with 10 μL of CCK-8 solution and incubated for 1 h. Cell viability was determined by measuring the absorbance at 450 nm. Each experiment was performed in triplicate.

Apoptosis of HSCs was assessed by fluorescein isothiocyanate (FITC)/propidium iodide (PI) staining. Briefly, HSCs (1×10^4 cells/well) were seeded into 24-well plates and exposed to different concentrations of ART (40 and 80 μM) for 48 h. Each well was supplemented with a mixture of 195 μL of Annexin V-FITC binding solution and 5 μL of Annexin V-FITC, followed by the addition of 10 μL of PI pigment. After incubation for 10-20 min in the dark at 20-25°C, the cells were observed under a fluorescence microscope.

Animal Model and ART Treatment

Forty clean-grade male Mongolian gerbils (weighing 80 g) were used in the experiment. *S. japonicum* cercariae were induced and collected by exposing infected water-immersed snails ~~with schistosomiasis~~ to light for 1.5 h and counted by direct observation under a light microscope. To establish the animal model of schistosomiasis, 30 Mongolian gerbils were infected with 80 *S. japonicum* cercariae

by the abdominal patch method. Praziquantel (PZQ), employed for decades as the key clinical agent to treat schistosomiasis, especially for adult worms, was used to eliminate the causative agent [18, 19]. Eight weeks post-infection, Mongolian gerbils infected with cercariae were administered PZQ diluted in 1% sodium carboxymethyl cellulose (CMC-Na) solution at a dose of 75 mg/kg·d^{-1} for 3 days via oral gavage. Twelve weeks post-infection, ~~30 cercariae-~~infected ~~Mongolian~~ gerbils were randomly divided into three groups: model infection group (1% CMC-Na, MOD), low-dose ART treatment group (20 mg/kg·d^{-1}, ART-L) and high-dose ART treatment group (40 mg/kg·d^{-1}, ART-H). Mongolian gerbils in ART-L and ART-H groups were treated with 0.2 mL ART solution with a corresponding concentration once daily for 12 weeks via oral gavage. Mongolian gerbils in normal control (CON) and MOD groups were administrated 0.2 mL 1% CMC-Na solution once daily for 12 weeks via oral gavage.

Measurement of Biochemical Indicators and Histological Assessment

An appropriate amount of liver tissue was ground with 9 times of tissue homogenate and then centrifuged at 3,500 rpm/min for 10 min. Then, 10% saline homogenization suspension was used to detect glutathione peroxidase (GPX), superoxide dismutase (SOD) and malondialdehyde (MDA) (Nanjing Jiancheng Bioengineering Institute, Nanjing, China) and hydroxyproline (Hyp) (Beijing Solarbio Science & Technology Co., Ltd, Beijing, China) using standard commercial kits according to the manufacturer’s instructions.

The superior segment of the left lateral lobe of each Mongolian gerbil liver was fixed in 10% formalin, embedded in paraffin, sectioned and stained with hematoxylin-eosin (H&E) and Masson’s trichrome for light microscopy. The Ishak scoring system was utilized to evaluate the degree of liver inflammation and fibrosis [20].

Whole Transcriptome Sequencing

Total RNA was isolated from liver tissue (n = 2) by Trizol regent (Invitrogen, CA, USA). Then, RNA concentration and purity were measured using Nanodrop 2000. RNA integrity was assessed using agarose gel electrophoresis and Bioanalyzer 2100 (Agilent, CA, USA), and RNA integrity number (RIN) >7.0 was considered sufficient for microarray analysis. A small RNA library was constructed using TruSeq Small RNA Sample Prep Kits (Illumina, San Diego, USA), and single-end sequencing was performed on Illumina Hi-seq 2500. For strand-specific library construction and paired-end sequencing on the Illumina Hi-seq 4000, RNA samples were purified using the Epicentre Ribo-Zero Gold Kit (Illumina, San Diego, USA) and fragmented into small pieces. The cleaved RNA fragments were then

reverse-transcribed to create the final complementary DNA (cDNA) library using the RNA-seq sample preparation kit (Illumina, San Diego, USA) according to the manufacturer's protocol.

Data Processing and Bioinformatics Analysis

Raw reads were processed by Cutadapt to generate clean reads and verified through FastQC (http://www.bioinformatics.babraham.ac.uk/projects/fastqc/). Clean reads were then mapped to the reference genome of the Mongolian gerbil and assembled using StringTie. Transcriptomes from Mongolian gerbil samples were merged to reconstruct a comprehensive transcriptome using Perl scripts. The transcript-level expression of RNA-seq was analyzed by calculating fragments per kilobase of exon per million mapped fragments (FPKM). DEGs with log2 fold change (FC) >1 or log2 FC < -1 and $p < 0.05$ were selected by the R package Ballgown. Gene ontology (GO) terms and the Kyoto encyclopedia of genes and genomes (KEGG) pathways of these DEGs were annotated. The protein-protein interaction (PPI) network of DEGs was performed using the STRING online analysis tool (https://string-db.org/). The PPI network was visualized and analyzed by Cytoscape software 3.7.2, and the top 30 DEGs were selected as hub genes. For microRNA (miRNA) analysis, unique sequences with 18-26 nucleotides were mapped to specific species precursors in miRBase 21.0 using the BLAST search to identify known miRNAs and novel 3p- and 5p-derived miRNAs. The prediction of miRNA target genes was carried out by OmicStudio tool at https://www.omicstudio.cn/tool based on TargetScan (5.0) and Miranda (3.3a), and the screening threshold was TargetScan_score ⩾50 and miranda_Energy < -10.

Quantitative PCR (qPCR) and Western Blot Assay

To validate RNA-seq results, total RNA was extracted from liver tissue using Trizol regent. Reverse transcription for each sample was performed on 200 ng of total RNA using a cDNA reverse transcription kit (Takara, Beijing, China). Thereafter, real-time fluorescent qPCR was utilized to detect the expression levels of the candidate genes. The primers were designed with Primer 5.0 software (**S1 Table**) and synthesized by Tsingke Biotechnology Co., Ltd (Beijing, China). The relative gene expression was calculated using the $2^{-\Delta\Delta}$Ct method and GAPDH was used as an internal reference gene for each example.

The total protein of the liver tissue was extracted using a total protein extraction kit. Total protein concentration was determined using the BCA kit (Sheng gong, Shanghai, China). Then, 30 µg of each protein sample was denatured at 100°C for 10 min and separated by sodium dodecyl-sulfate polyacrylamide gel electrophoresis (SDS-PAGE). Protein samples were then transferred to polyvinylidene difluoride membranes, blocked with 5% non-fatty milk for 1 h and blotted with relative antibodies. The enhanced chemiluminescence reagent was used for color

development. The Alpha software processing system was used to analyze the optical density values of the target band.

Statistics Analysis

Data were presented as the mean ± SEM. Statistical analyses were performed using SPSS 26.0 and Graph Pad Prism 8.4 software. Statistical comparison between groups was performed using one-way analysis of variance (ANOVA) with a Tukey test and student's t-test. $p < 0.05$ was considered statistically significant.

Results

Effect of ART on HSCs and the Animal Model

The effect of ART on HSCs was investigated using CCK-8 and FITC/PI double staining assays. After ART treatment, apoptotic morphological changes were observed under the microscope. Results showed that ART (10 μM) significantly inhibited HSCs proliferation at 24 h after treatment ($p < 0.05$ vs. control) (**Fig 1A and B**). Meanwhile, the number of apoptotic cells gradually increased with the increase in ART concentration (**Fig 1C**). **Figure 2A** shows the respective timeline of the *in vivo* studies. After 12 weeks of ART treatment, Hyp content decreased significantly ($p < 0.01$) and GPX activity increased significantly in the ART-H group compared to the MOD group. While changes in MDA content and SOD activity did not show a statistical significance (**Fig 2B**). The livers in the MOD group were chestnut brown and had slightly increased volume and blunt edges. There was a marked improvement in liver morphology and color in the ART-H group; however, changes to the liver index were not significant (**S2 Table**). Histopathological changes in livers were assessed by H&E and Masson's trichrome staining. Compared with the CON group, inflammatory cell infiltration and collagen fiber deposition increased significantly in the MOD group ($p < 0.05$), while collagen deposition decreased significantly in the ART-H group, indicating that 40 μM of ART have a significant protective effect against liver fibrosis ($p < 0.01$) (**Fig 2C**).

Fig 1. Effect of ART on HSCs. (A) HSCs morphology after 24 h of treatment with different ART concentrations under an inverted phase-contrast microscope (100× magnification). (B) Viability of HSCs incubated with different concentrations of ART for 24 h. (C) Fluorescence microscopy images of apoptotic cells after 24 h of treatment with different concentrations of ART, at 100× magnification. Data are presented as mean ± SEM (n = 3). $^{***}p < 0.001$ vs CON group.

Fig 2. Effect of ART on schistosomiasis-induced liver fibrosis in the Mongolian gerbil. (A) Timeline of *in vivo* studies. (B) Levels of Hyp, MDA, SOD, and GPX. (C) Liver morphological and pathological changes of liver tissues (100× magnification),

hematoxylin-eosin (H&E) and Masson staining score statistics. Data are presented as mean ± SEM (n = 3). ** $p< 0.01$ and *** $p <0.001$ vs CON group. $^{\#}$ $p< 0.05$ vs MOD group)

Transcriptome Expression Profiles of Schistosomiasis-Induced Liver Fibrosis in Response to ART

For each sequencing library, the reads of the Q20, Q30 and GC content exceeded 99%, 96% and 47% respectively, which indicated a high quality of clean data (**S3 Table**). Meanwhile, over 90% of the total reads were successfully mapped to the reference genome (**S4 Table**). Successfully mapped reads were then used for subsequent analysis. DEGs were then screened for subsequent analysis. Compared with the CON group, 467 genes were up-regulated and 144 genes were down-regulated in the MOD group, while 187 genes were up-regulated and 142 genes were down-regulated in the ART group. Compared ART group with the MOD group, 117 genes were up-regulated and 216 genes were down-regulated in the ART group (**Fig 3A**). The Venn diagram showed the shared and unique DEGs between different groups. Except for ART vs CON, DEGs between MOD vs CON and ART vs MOD were considered ART treatment-related genes, which revealed 158 DEGs related to the ART effect against schistosomiasis-induced liver fibrosis (**Fig 3B**). The heat map revealed differential genes expression patterns between different groups (**Fig 3C**). Meanwhile, a total of 44 differentially expressed miRNAs related to ART-induced anti-schistosomiasis liver fibrosis were identified **(S1 Fig)**. The gene and protein expression levels of pro-fibrosis factors such as Ctgf, α-SMA, TGF-β1, Vim, and collagen detected by qPCR and western blot were consistent with sequencing results, indicating that sequencing results are reliable (**Fig 3D**).

Fig 3. Screening and validation of DEGs. (A) Volcano plot, (B) Venn diagram and (C) Heat map of DEGs. (D) qPCR validation of DEGs. (E) Western blot validation of DEGs. Data are presented as mean ± SEM (n = 2). ** $p< 0.01$ and *** $p< 0.001$ vs CON group; $^{\#\#\#}$ $p< 0.001$ vs MOD group.

Enrichment Analysis of DEGs and PPI Network Construction

Subsequently, GO and KEGG enrichment analyses of 158 DEGs were performed respectively to explore the potential mechanism of ART. The top 20 significant GO and KEGG pathway terms are displayed in **Fig 4A and B**. GO analysis showed that these DEGs were mainly enriched in immune response, inflammatory response and integrin-mediated signaling pathways. (**Fig 4A**). KEGG pathway analysis revealed that these DEGs were mainly enriched in complement and coagulation cascades,

chemokine signaling pathway, FC gamma R-mediated phagocytosis, focal adhesion, platelet activation, toll-like receptor signaling pathway and HIF-1 signaling pathway (**Fig 4B**). The PPI network of the 158 DEGs generated 109 nodes and 271 edges (p< 1.0-16), and the top 30 DEGs were selected as hub genes (**Fig 4C**). Most of the predicted target genes of 6 miRNAs (miR-193a-3p, miR-370-3p, miR-365-3p, miR-2137, miR-3473b and miR-3473e) were also differentially expressed hub genes related to ART-induced anti-schistosomiasis liver fibrosis, including Tlr4, Cyth4, Irf8, Nckap1l, Myo1F, Src, Fscn1, Adamtsl2, Prkcd, Surf4 and Ddit4 (**S5 Table**). These DEGs were implicated in key pathways, such as immune and inflammatory response, integrin-mediated signaling and toll-like receptor signaling pathways.

Fig 4. Enrichment analysis of DEGs and PPI network construction. (A) GO enrichment analysis. (B) KEGG pathway enrichment analysis. (C) a, PPI analysis; blue represents down-regulated genes, and red represents up-regulated genes. b, Top 30 hub genes based on the degree algorithm of cytoHubba; dark red represents a high degree of connectivity.

Fig 5. Potential candidate regulators of schistosomiasis-induced liver fibrosis in response to ART therapy. Red arrows indicate up-regulated genes and green arrows indicate down-regulated genes; an asterisk indicates previously reported genes. “X” indicates blockage of the fibrotic process. ART may alleviate liver fibrosis in the gerbil model by reducing inflammation, inhibiting HSCs activation and decreasing collagen deposition. Liver fibrosis induced by *Schistosoma japonicum* infection is the result of the deposition of eggs in the liver. Matured eggs cause blood vessel and hepatocyte damage by SEA, which triggers egg granuloma with a complex immune inflammatory reaction regulated by damaged hepatocytes, thereby recruiting immune cells. ART reduced liver inflammation possibly by up-regulating miR-193-3p that may inhibit TLR4 signaling, and inhibiting producing profibrotic cytokines such as TGF-β1, thereby inhibiting HSCs activation. ART may also induce apoptosis of activated HSCs through mitochondrial pathways. It possibly decreases the ECM deposition by down-regulating genes associated with collagen synthesis such as Ctgf, Qsox1 and Itgb2.

Discussion

Schistosomiasis — mostly caused by infection with *S. japonicum* — mainly leads to liver fibrosis and advanced diseases, such as portal hypertension [21]. PZQ has been approved for the treatment of schistosomiasis because of its high effectiveness. Oral administration of PZQ at a single dose of 400 mg/kg resulted in worm reduction rates of 88-100% in mice [22], which indicated that our dose (75 mg/kg for 3 days) can effectively kill worms. Additionally, ART can effectively kill juvenile schistosomulum and has a very good effect of preventing schistosomiasis, so it will not cause the survival and expulsion of eggs [23]. However, previous studies demonstrated that the removal or elimination of the etiological agents may not be sufficient to stop liver fibrosis progression [24].

ART has received considerable attention because of its various pharmacological properties. Although the antifibrotic effect of ART has been proven, the specific molecular mechanism has not been systematically elucidated [25]. The present study investigated the effect of ART on liver fibrosis in HSCs and the *S. japonicum*-infected Mongolian gerbil model. Mechanistically, RNA-seq analysis of liver tissue was performed to explore the potential molecular mechanism of ART in liver fibrosis.

Our data showed that ART significantly inhibited the proliferation and induced the apoptosis of HSCs *in vitro*, and effectively alleviated schistosomiasis-induced liver fibrosis *in vivo*. In HSCs, ART significantly inhibited cell proliferation in a dose-dependent manner in a concentration range of 10-20 μM. In addition, the number of apoptotic cells gradually increased with the increase in the concentration of ART, indicating that ART promotes cell apoptosis in a dose-dependent manner. A recent study also showed that ART promotes HSCs apoptosis by inhibiting mitochondrial function [14]. Moreover, we successfully ~~established~~ a Mongolian gerbil model of liver fibrosis induced by *S. japonicum* cercariae infection to explore the antifibrotic activity of ART *in vivo*. A study on the acute toxicity of intravenous ART in rats showed that the LD50 value was 488 mg/kg in rats [26]. Our previous long-term toxicity study showed that the LD50 of artesunate given orally to rats once daily for 28 d was 150 mg/kg/d and the safe dose was 25 mg/kg/d [27]. Therefore, after conversion, we chose a high dose of 40 mg/kg, which is relatively safe in animal experiments. It was found that gavage ATR treatment slightly improved the liver index and reduced inflammatory cell infiltration and collagen deposition in the ART-H group, which is consistent with findings of a previous study that reported a decreased area of fibrosis and granuloma in the ART intervention group in a mouse model [14]. Studies have shown that ART is effective against juvenile schistosomulum, and has a very good effect of preventing schistosomiasis, so it will not cause the survival and expulsion of eggs [23]. Also, no live adult worms or eggs were found by tissue biopsy before ART administration. In addition, PZQ did not reduce fibrosis in the MOD group, which indicates that the reduction of fibrosis in the ART group was due to its antifibrotic effect rather than its antimalarial effect. Collectively, these results suggest that ART may relieve schistosomiasis-induced liver fibrosis to a certain extent.

To further explore the antifibrotic mechanism of ART, we performed transcriptome RNA-seq of the liver from CON, MOD and ART groups. Further, bioinformatics analysis identified 158 DEGs related to ART-induced anti-schistosomiasis liver fibrosis. GO function analysis showed that these DEGs were involved in immune response, extracellular space and calcium ion transportation terms. KEGG pathway analysis revealed that the DEGs were significantly enriched in immune response, focal adhesion, platelet activation and HIF-1 signaling pathways.

Current treatment strategies for liver fibrosis directly inhibit HSCs activation, induce activated HSCs apoptosis, increase ECM degradation and suppress inflammation (**Fig 5**). The activation of HSCs is crucial in liver fibrosis, and TGF-β1

is responsible for HSCs activation. The Src family of tyrosine kinases are activated in fibrosis of various organs such as the lung, kidney and liver, and are considered to be the amplifiers of TGF-β1 signal in the pathological process of fibrosis [28]. The activated Src under hypoxic conditions of tissue cells can mediate the expression of Ctgf induced by TGF-β1, which further stimulates collagen secretion, eventually accelerating the fibrotic process [29, 30]. At present, Src has become a potential therapeutic target for various organ fibrosis. For example, Sarakatinib, a tyrosine kinase activity inhibitor, has been approved by the Food and Drug Administration for the treatment of idiopathic pulmonary fibrosis [31, 32]. Our RNA-seq results showed that Src was significantly down-regulated in the ART group compared with the MOD group, which indicated that Src is a potential target of ART in liver fibrosis [33]. Additionally, previous studies reported that ART prevents schistosomiasis-induced liver fibrosis by regulating mitochondrial function in HSCs [7, 14]. Six mitochondrial-related DEGs (Ddit4, Ndufa7, Higd2a, Ucp2, Mrpl36 and Fahd1) were identified in the present study, which are potential targets of ART. The Ddit4 is expressed under stress and is located downstream of HIF-1α, which negatively regulates the mammalian target of rapamycin (mTOR)-induced cell proliferation and induces autophagy [34, 35]. Ctss is one of the top 30 DEGs identified in this study. It promotes cell migration and invasion by regulating Ca^{2+} homeostasis and ECM degradation [36]. Studies have shown that inhibiting Ctss may increase the expression of mitochondrial calcium uniporter and enhance the absorption capacity of mitochondrial Ca^{2+}, leading to mitochondrial Ca^{2+} overload, the production of large amounts of ROS and mitochondria-mediated cell apoptosis [37]. However, further *in vivo* experimental verification is required to confirm this interesting hypothesis.

Excessive deposition of ECM is the main pathological feature of liver fibrosis, and thus increasing ECM degradation is a pivotal strategy for antifibrotic therapies. RNA-seq and PPI analysis showed that several DEGs, such as Ctgf, Qsox1, Fscn1, Myo1f, Lpxn, Scin, Parvg, Itgb2 and Fermt3, may participate in the regulation of actin cytoskeleton assembly and ECM adhesion [38-41]. Interestingly, Ctgf has been reported to be up-regulated in various fibrotic diseases, including lung, kidney, skin and liver fibrosis [42, 43]. Inhibition of Ctgf inactivated HSCs and reduced ECM formation [44, 45]. Therefore, Ctgf may be a potential therapeutic target for reversing liver fibrosis.

Conclusion

In summary, we successfully ~~established~~ a Mongolian gerbil model of liver fibrosis induced by *S. japonicum* cercariae infection, mimicking human infection. The efficacy test showed that ART effectively prevented liver fibrosis *in vitro* and *in vivo*. ART may effectively alleviate schistosomiasis-induced liver fibrosis by reducing inflammation and collagen fiber deposition. We established the first comprehensive transcription profile, mined host candidate regulators and provided an overview of the complex network of the mechanism of schistosomiasis-induced liver fibrosis in response to ART. The findings of this study are essential to

understanding the mechanism of action of ART and verifying potential diagnostic markers or novel therapeutic agents for schistosomiasis-induced liver fibrosis.

Conflict of interest

The authors declare that the research was conducted in the absence of any commercial or financial relationships that could be construed as a potential conflict of interest.

Author contributions

Conceptualization: Qingming Kong, Yajie Yuan.
Data curation: Yajie Yuan, Xinyue Lv.
Funding acquisition: Qingming Kong, Fangwei Dai, Haojie Ding.
Investigation: Qingming Kong.
Methodology: Wenxia Zhao, Qunbo Tong, Jianzu Ding, Di Lou, Xiaofeng Chu.
Project administration: Qingming Kong.
Resources: Fangwei Dai.
Supervision: Qingming Kong, Shaohong Lu.
Validation: Haojie Ding, Riping Chen, Bin Zheng.
Visualization: Yajie Yuan, Xinyue Lv.
Writing –original draft: Yajie Yuan.
Writing –review & editing: Qingming Kong, Yajie Yuan, Xinyue Lv, Shaohong Lu.

Funding

This study was supported by the National Natural Science Foundation of China (31501050), Zhejiang Provincial Program for the Cultivation of High-Level Innovative Health Talents (WJW2021002), Basic Public Welfare Research Project of Zhejiang Province (LGF22H180015), Medical Health Science and Technology Project of Zhejiang Provincial Health Commission (2020KY530) and the Zhejiang Provincial Natural Science Foundation of China (LQY19H190002).

Supplementary Information and Data Availability

Tables S1-S5 and Fig. S1 can be found in the Supporting Information files. Raw RNA sequencing data were uploaded to the Sequencing Read Archive of the National Center for Biotechnology Information (NCBI) (Project number PRJNA846574).

Reference

1. Friedman SL. Hepatic fibrosis -- overview. Toxicology. 2008;254(3):120-9. Epub 2008/07/30. doi: 10.1016/j.tox.2008.06.013. PubMed PMID: 18662740.
2. Lee YA, Wallace MC, Friedman SL. Pathobiology of liver fibrosis: a translational success story. Gut. 2015;64(5):830-41. Epub 2015/02/15. doi: 10.1136/gutjnl-2014-306842. PubMed PMID:

25681399; PubMed Central PMCID: PMCPMC4477794.

3. Chen RJ, Wu HH, Wang YJ. Strategies to prevent and reverse liver fibrosis in humans and laboratory animals. Arch Toxicol. 2015;89(10):1727-50. Epub 2015/05/13. doi: 10.1007/s00204-015-1525-6. PubMed PMID: 25963329.

4. Mahdinloo S, Kiaie SH, Amiri A, Hemmati S, Valizadeh H, Zakeri-Milani P. Efficient drug and gene delivery to liver fibrosis: rationale, recent advances, and perspectives. Acta Pharm Sin B. 2020;10(7):1279-93. Epub 2020/09/03. doi: 10.1016/j.apsb.2020.03.007. PubMed PMID: 32874828; PubMed Central PMCID: PMCPMC7451940.

5. Chen J, Hu Y, Chen L, Liu W, Mu Y, Liu P. The effect and mechanisms of Fuzheng Huayu formula against chronic liver diseases. Biomed Pharmacother. 2019;114:108846. Epub 2019/04/10. doi: 10.1016/j.biopha.2019.108846. PubMed PMID: 30965233.

6. Tian H, Liu L, Li Z, Liu W, Sun Z, Xu Y, et al. Chinese medicine CGA formula ameliorates liver fibrosis induced by carbon tetrachloride involving inhibition of hepatic apoptosis in rats. J Ethnopharmacol. 2019;232:227-35. Epub 2018/11/25. doi: 10.1016/j.jep.2018.11.027. PubMed PMID: 30471378.

7. Luo JT, Qian SN, Wu KY, Zhu SC, Huang YL, Ye JP, et al. [Exploring the effects of artesunate and fuzheng huayu decoction on mitochondria in the treatment of schistosomiasis liver fibrosis]. Zhonghua Gan Zang Bing Za Zhi. 2022;30(1):45-51. Epub 2021/02/26. doi: 10.3760/cma.j.cn501113-20201024-00577. PubMed PMID: 33626860.

8. Vatsveen TK, Myhre MR, Steen CB, Walchli S, Lingjaerde OC, Bai B, et al. Artesunate shows potent anti-tumor activity in B-cell lymphoma. J Hematol Oncol. 2018;11(1):23. Epub 2018/02/21. doi: 10.1186/s13045-018-0561-0. PubMed PMID: 29458389; PubMed Central PMCID: PMCPMC5819282.

9. Yan G, Dawood M, Bockers M, Klauck SM, Fottner C, Weber MM, et al. Multiple modes of cell death in neuroendocrine tumors induced by artesunate. Phytomedicine. 2020;79:153332. Epub 2020/09/22. doi: 10.1016/j.phymed.2020.153332. PubMed PMID: 32957040.

10. Zeng XZ, Zhang YY, Yang Q, Wang S, Zou BH, Tan YH, et al. Artesunate attenuates LPS-induced osteoclastogenesis by suppressing TLR4/TRAF6 and PLCgamma1-Ca(2+)-NFATc1 signaling pathway. Acta Pharmacol Sin. 2020;41(2):229-36. Epub 2019/08/23. doi: 10.1038/s41401-019-0289-6. PubMed PMID: 31431733; PubMed Central PMCID: PMCPMC7468527.

11. Lai L, Chen Y, Tian X, Li X, Zhang X, Lei J, et al. Artesunate alleviates hepatic fibrosis induced by multiple pathogenic factors and inflammation through the inhibition of LPS/TLR4/NF-kappaB signaling pathway in rats. Eur J Pharmacol. 2015;765:234-41. Epub 2015/09/01. doi: 10.1016/j.ejphar.2015.08.040. PubMed PMID: 26318197.

12. Lv J, Bai R, Wang L, Gao J, Zhang H. Artesunate may inhibit liver fibrosis via the FAK/Akt/beta-catenin pathway in LX-2 cells. BMC Pharmacol Toxicol. 2018;19(1):64. Epub 2018/10/18. doi: 10.1186/s40360-018-0255-9. PubMed PMID: 30326962; PubMed Central PMCID: PMCPMC6192352.

13. Kong Z, Liu R, Cheng Y. Artesunate alleviates liver fibrosis by regulating ferroptosis signaling pathway. Biomed Pharmacother. 2019;109:2043-53. Epub 2018/12/16. doi: 10.1016/j.biopha.2018.11.030. PubMed PMID: 30551460.

14. Shen S, Luo J, Ye J. Artesunate alleviates schistosomiasis-induced liver fibrosis by downregulation of mitochondrial complex subunit NDUFB8 and complex subunit UQCRC2 in

hepatic stellate cells. Acta Trop. 2021;214:105781. Epub 2020/12/03. doi: 10.1016/j.actatropica.2020.105781. PubMed PMID: 33264632.
15. Liang YR, Guo YX, Yi XY, Zeng XF, Peng LX, Zeng QS. Schistosoma japonicum: a comparison of the development of the parasite and associated pathological changes in mice and jirds (Meriones unguiculatus). Int J Parasitol. 1983;13(6):531-8. Epub 1983/12/01. doi: 10.1016/s0020-7519(83)80024-1. PubMed PMID: 6654579.
16. Dunn MA, Cheever AW, Paglia LM, Kelly EP, Duvall RH, Andrade ZA, et al. Reversal of advanced liver fibrosis in rabbits with Schistosomiasis japonica. Am J Trop Med Hyg. 1994;50(4):499-505. Epub 1994/04/01. doi: 10.4269/ajtmh.1994.50.499. PubMed PMID: 8166357.
17. Xu L, Hui AY, Albanis E, Arthur MJ, O'Byrne SM, Blaner WS, et al. Human hepatic stellate cell lines, LX-1 and LX-2: new tools for analysis of hepatic fibrosis. Gut. 2005;54(1):142-51. Epub 2004/12/14. doi: 10.1136/gut.2004.042127. PubMed PMID: 15591520; PubMed Central PMCID: PMCPMC1774377.
18. Pica-Mattoccia L, Cioli D. Sex- and stage-related sensitivity of Schistosoma mansoni to in vivo and in vitro praziquantel treatment. Int J Parasitol. 2004;34(4):527-33. Epub 2004/03/12. doi: 10.1016/j.ijpara.2003.12.003. PubMed PMID: 15013742.
19. Koehne E, Zander N, Rodi M, Held J, Hoffmann W, Zoleko-Manego R, et al. Evidence for in vitro and in vivo activity of the antimalarial pyronaridine against Schistosoma. PLoS Negl Trop Dis. 2021;15(6):e0009511. Epub 2021/06/25. doi: 10.1371/journal.pntd.0009511. PubMed PMID: 34166393; PubMed Central PMCID: PMCPMC8263063.
20. Ishak K, Baptista A, Bianchi L, Callea F, De Groote J, Gudat F, et al. Histological grading and staging of chronic hepatitis. J Hepatol. 1995;22(6):696-9. Epub 1995/06/01. doi: 10.1016/0168-8278(95)80226-6. PubMed PMID: 7560864.
21. Gryseels B, Polman K, Clerinx J, Kestens L. Human schistosomiasis. Lancet. 2006;368(9541):1106-18. Epub 2006/09/26. doi: 10.1016/S0140-6736(06)69440-3. PubMed PMID: 16997665.
22. Xiao SH, Sun J, Chen MG. Pharmacological and immunological effects of praziquantel against Schistosoma japonicum: a scoping review of experimental studies. Infect Dis Poverty. 2018;7(1):9. Epub 2018/02/08. doi: 10.1186/s40249-018-0391-x. PubMed PMID: 29409536; PubMed Central PMCID: PMCPMC5801800.
23. Gouveia MJ, Brindley PJ, Gartner F, Costa J, Vale N. Drug Repurposing for Schistosomiasis: Combinations of Drugs or Biomolecules. Pharmaceuticals (Basel). 2018;11(1). Epub 2018/02/07. doi: 10.3390/ph11010015. PubMed PMID: 29401734; PubMed Central PMCID: PMCPMC5874711.
24. Wang FD, Zhou J, Chen EQ. Molecular Mechanisms and Potential New Therapeutic Drugs for Liver Fibrosis. Front Pharmacol. 2022;13:787748. Epub 2022/03/01. doi: 10.3389/fphar.2022.787748. PubMed PMID: 35222022; PubMed Central PMCID: PMCPMC8874120.
25. Wang Y, Wang Y, You F, Xue J. Novel use for old drugs: The emerging role of artemisinin and its derivatives in fibrosis. Pharmacol Res. 2020;157:104829. Epub 2020/05/04. doi: 10.1016/j.phrs.2020.104829. PubMed PMID: 32360483.
26. Li Q, Xie LH, Johnson TO, Si Y, Haeberle AS, Weina PJ. Toxicity evaluation of artesunate and artelinate in Plasmodium berghei-infected and uninfected rats. Trans R Soc Trop Med Hyg. 2007;101(2):104-12. Epub 2006/07/25. doi: 10.1016/j.trstmh.2006.04.010. PubMed PMID: 16860356.
27. Guo Y WL, Xu PS, Yang MJ, Fan JT, Li SW. Safety assessment for long-term oral treatment

with artesunate in rats. Zhongguo Xue Xi Chong Bing Fang Zhi Za Zhi. 1998;12(1):17-9. (in Chinese). doi: 10.16250/j.32.1374.2000.01.015.

28. Wang J, Zhuang S. Src family kinases in chronic kidney disease. Am J Physiol Renal Physiol. 2017;313(3):F721-F8. Epub 2017/06/16. doi: 10.1152/ajprenal.00141.2017. PubMed PMID: 28615246; PubMed Central PMCID: PMCPMC5625110.

29. Mishra R, Zhu L, Eckert RL, Simonson MS. TGF-beta-regulated collagen type I accumulation: role of Src-based signals. Am J Physiol Cell Physiol. 2007;292(4):C1361-9. Epub 2006/12/01. doi: 10.1152/ajpcell.00370.2006. PubMed PMID: 17135298.

30. Cheng Z, Liu L, Wang Z, Cai Y, Xu Q, Chen P. Hypoxia Activates Src and Promotes Endocytosis Which Decreases MMP-2 Activity and Aggravates Renal Interstitial Fibrosis. Int J Mol Sci. 2018;19(2). Epub 2018/02/22. doi: 10.3390/ijms19020581. PubMed PMID: 29462885; PubMed Central PMCID: PMCPMC5855803.

31. Li LF, Kao KC, Liu YY, Lin CW, Chen NH, Lee CS, et al. Nintedanib reduces ventilation-augmented bleomycin-induced epithelial-mesenchymal transition and lung fibrosis through suppression of the Src pathway. J Cell Mol Med. 2017;21(11):2937-49. Epub 2017/06/10. doi: 10.1111/jcmm.13206. PubMed PMID: 28598023; PubMed Central PMCID: PMCPMC5661114.

32. Liu F, Wang L, Qi H, Wang J, Wang Y, Jiang W, et al. Nintedanib, a triple tyrosine kinase inhibitor, attenuates renal fibrosis in chronic kidney disease. Clin Sci (Lond). 2017;131(16):2125-43. Epub 2017/06/25. doi: 10.1042/CS20170134. PubMed PMID: 28646122.

33. Berkoz M, Ozkan-Yilmaz F, Ozluer-Hunt A, Krosniak M, Turkmen O, Korkmaz D, et al. Artesunate inhibits melanoma progression in vitro via suppressing STAT3 signaling pathway. Pharmacol Rep. 2021;73(2):650-63. Epub 2021/02/21. doi: 10.1007/s43440-021-00230-6. PubMed PMID: 33609273.

34. Chen R, Wang B, Chen L, Cai D, Li B, Chen C, et al. DNA damage-inducible transcript 4 (DDIT4) mediates methamphetamine-induced autophagy and apoptosis through mTOR signaling pathway in cardiomyocytes. Toxicol Appl Pharmacol. 2016;295:1-11. Epub 2016/01/31. doi: 10.1016/j.taap.2016.01.017. PubMed PMID: 26825372.

35. Gharibi B, Ghuman M, Hughes FJ. DDIT4 regulates mesenchymal stem cell fate by mediating between HIF1alpha and mTOR signalling. Sci Rep. 2016;6:36889. Epub 2016/11/24. doi: 10.1038/srep36889. PubMed PMID: 27876894; PubMed Central PMCID: PMCPMC5120275.

36. Lin HH, Chen SJ, Shen MR, Huang YT, Hsieh HP, Lin SY, et al. Lysosomal cysteine protease cathepsin S is involved in cancer cell motility by regulating store-operated Ca(2+) entry. Biochim Biophys Acta Mol Cell Res. 2019;1866(12):118517. Epub 2019/07/25. doi: 10.1016/j.bbamcr.2019.07.012. PubMed PMID: 31340164.

37. Fei M, Zhang L, Wang H, Zhu Y, Niu W, Tang T, et al. Inhibition of Cathepsin S Induces Mitochondrial Apoptosis in Glioblastoma Cell Lines Through Mitochondrial Stress and Autophagosome Accumulation. Front Oncol. 2020;10:516746. Epub 2021/01/12. doi: 10.3389/fonc.2020.516746. PubMed PMID: 33425712; PubMed Central PMCID: PMCPMC7787074.

38. Gupta A, Lee BS, Khadeer MA, Tang Z, Chellaiah M, Abu-Amer Y, et al. Leupaxin is a critical adaptor protein in the adhesion zone of the osteoclast. J Bone Miner Res. 2003;18(4):669-85. Epub 2003/04/04. doi: 10.1359/jbmr.2003.18.4.669. PubMed PMID: 12674328.

39. Yoshimi R, Yamaji S, Suzuki A, Mishima W, Okamura M, Obana T, et al. The gamma-parvin-integrin-linked kinase complex is critically involved in leukocyte-substrate interaction.

J Immunol. 2006;176(6):3611-24. Epub 2006/03/07. doi: 10.4049/jimmunol.176.6.3611. PubMed PMID: 16517730.

40. Navines-Ferrer A, Martin M. Long-Tailed Unconventional Class I Myosins in Health and Disease. Int J Mol Sci. 2020;21(7). Epub 2020/04/11. doi: 10.3390/ijms21072555. PubMed PMID: 32272642; PubMed Central PMCID: PMCPMC7177449.

41. Ou C, Sun Z, He X, Li X, Fan S, Zheng X, et al. Targeting YAP1/LINC00152/FSCN1 Signaling Axis Prevents the Progression of Colorectal Cancer. Adv Sci (Weinh). 2020;7(3):1901380. Epub 2020/02/12. doi: 10.1002/advs.201901380. PubMed PMID: 32042551; PubMed Central PMCID: PMCPMC7001651.

42. Gressner OA, Gressner AM. Connective tissue growth factor: a fibrogenic master switch in fibrotic liver diseases. Liver Int. 2008;28(8):1065-79. Epub 2008/09/12. doi: 10.1111/j.1478-3231.2008.01826.x. PubMed PMID: 18783549.

43. Shi C, Li G, Tong Y, Deng Y, Fan J. Role of CTGF gene promoter methylation in the development of hepatic fibrosis. Am J Transl Res. 2016;8(1):125-32. Epub 2016/04/14. PubMed PMID: 27069546; PubMed Central PMCID: PMCPMC4759422.

44. Yuhua Z, Wanhua R, Chenggang S, Jun S, Yanjun W, Chunqing Z. Disruption of connective tissue growth factor by short hairpin RNA inhibits collagen synthesis and extracellular matrix secretion in hepatic stellate cells. Liver Int. 2008;28(5):632-9. Epub 2008/04/25. doi: 10.1111/j.1478-3231.2008.01730.x. PubMed PMID: 18433392.

45. Hao C, Xie Y, Peng M, Ma L, Zhou Y, Zhang Y, et al. Inhibition of connective tissue growth factor suppresses hepatic stellate cell activation in vitro and prevents liver fibrosis in vivo. Clin Exp Med. 2014;14(2):141-50. Epub 2013/03/05. doi: 10.1007/s10238-013-0229-6. PubMed PMID: 23456538.

Supporting information

S1 Fig. Differentially expressed miRNAs. (A) Heat map of differentially expressed miRNAs and (B) Venn diagram of differentially expressed miRNAs.

S1 Table. Primer information.

S2 Table. Liver mass index.

S3 Table. Quality parameters of clean data.

S4 Table. Number of successfully mapped reads.

S5 Table: MiRNAs target gene prediction results.

Click here to access/download;Figure;Fig 1.tif

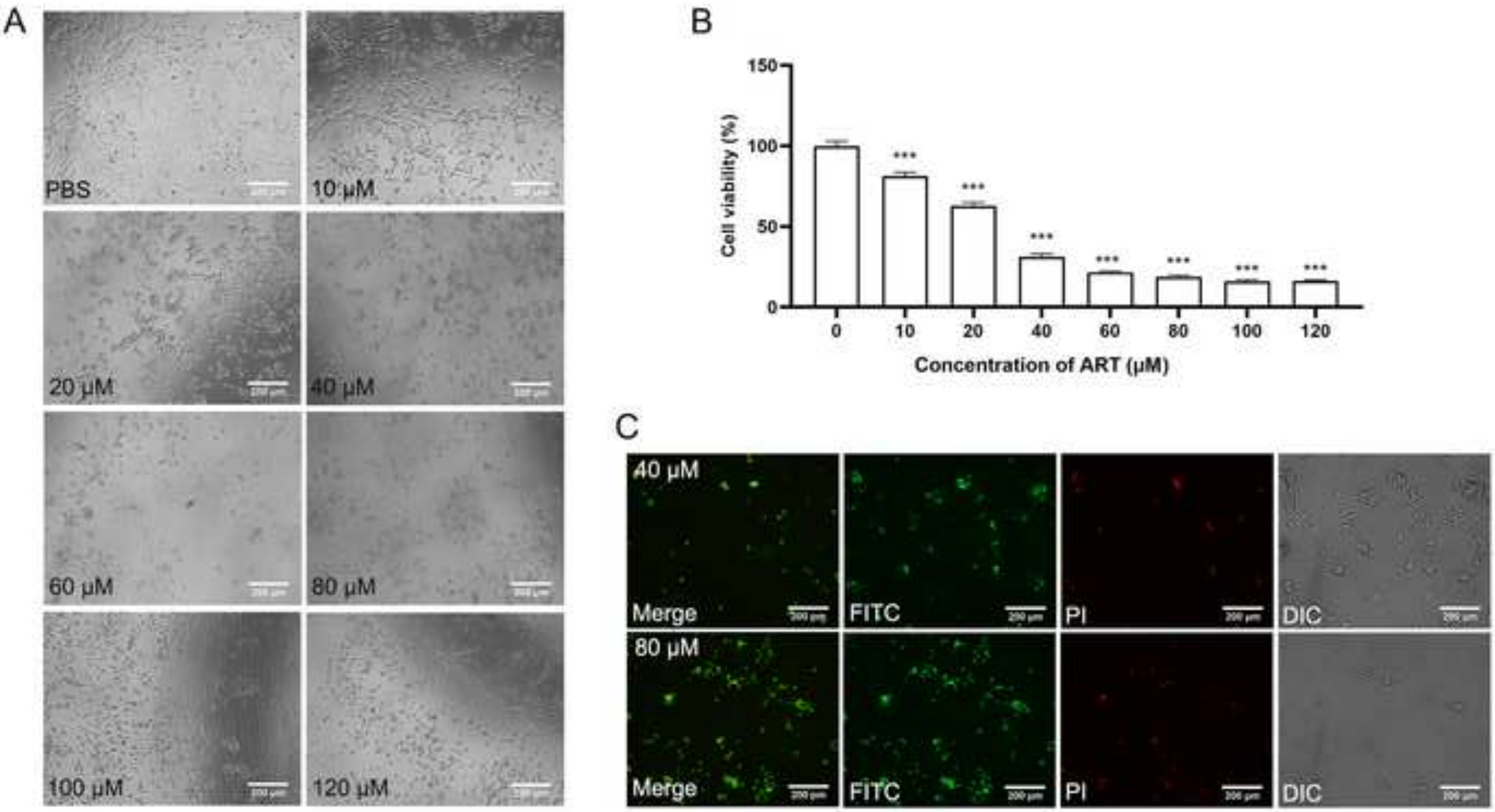

Figure

Click here to access/download;Figure;Fig 2.tif

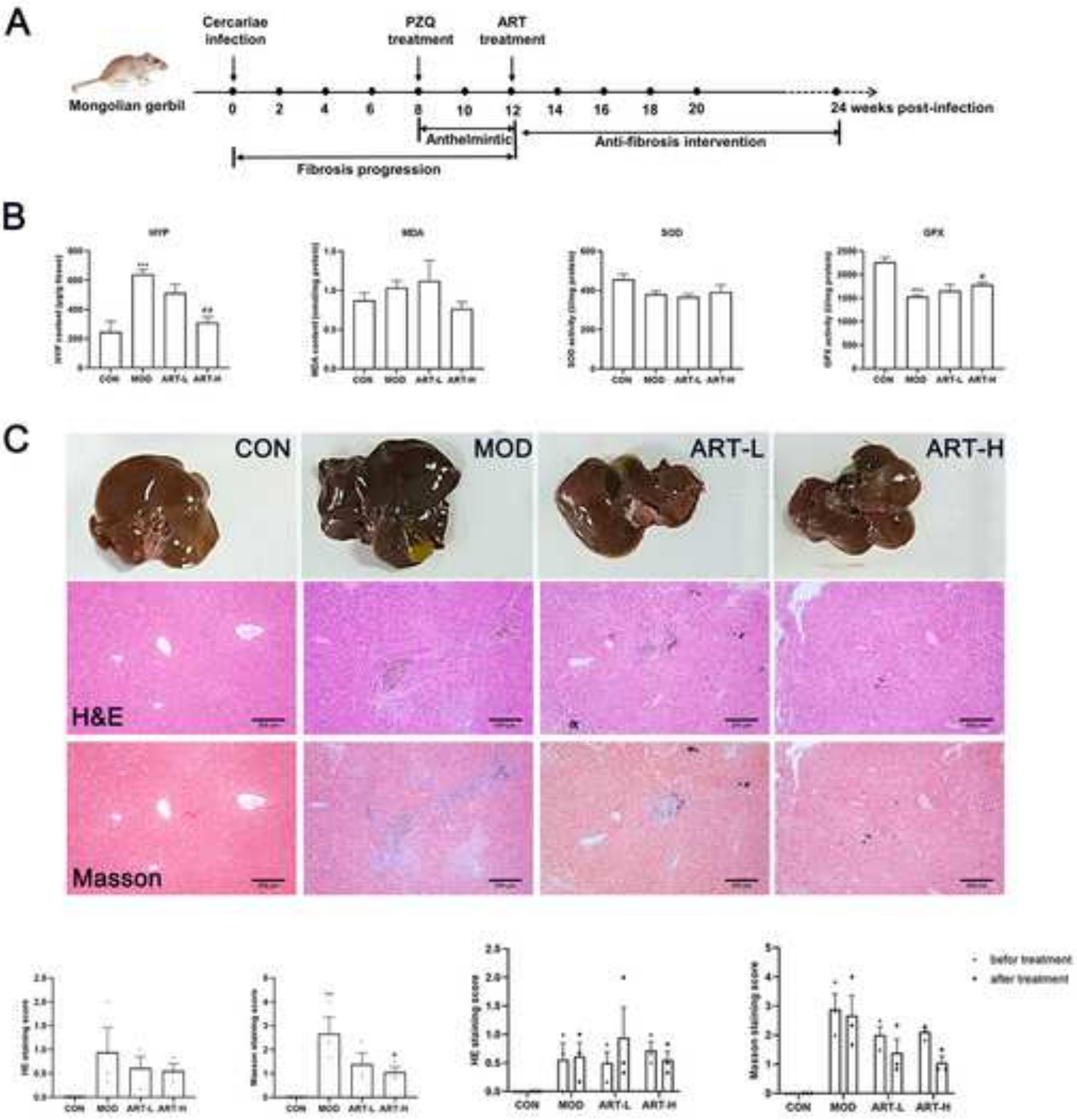

Figure

Click here to access/download;Figure;Fig 3.tif

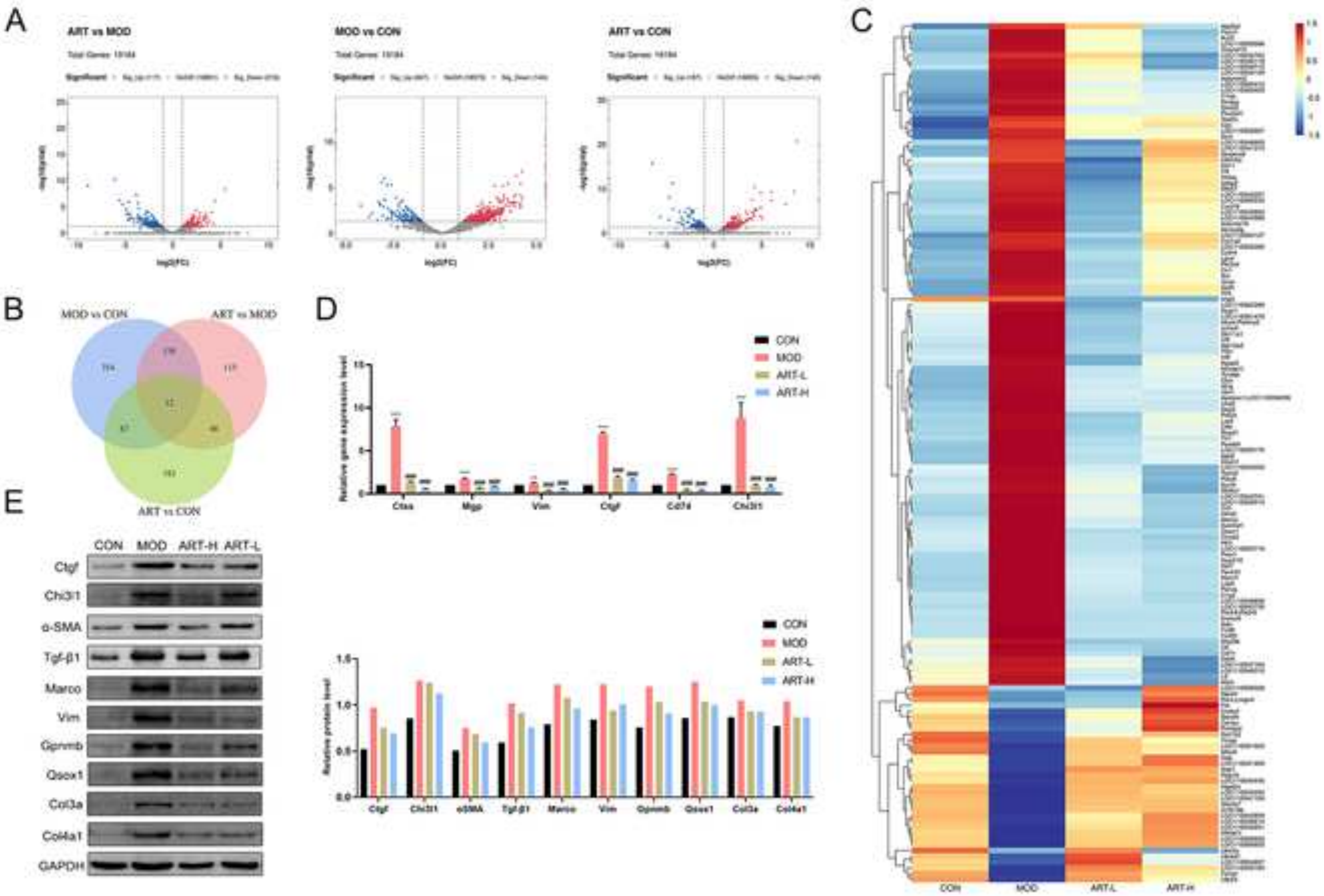

Figure

Click here to access/download;Figure;Fig 4.tif

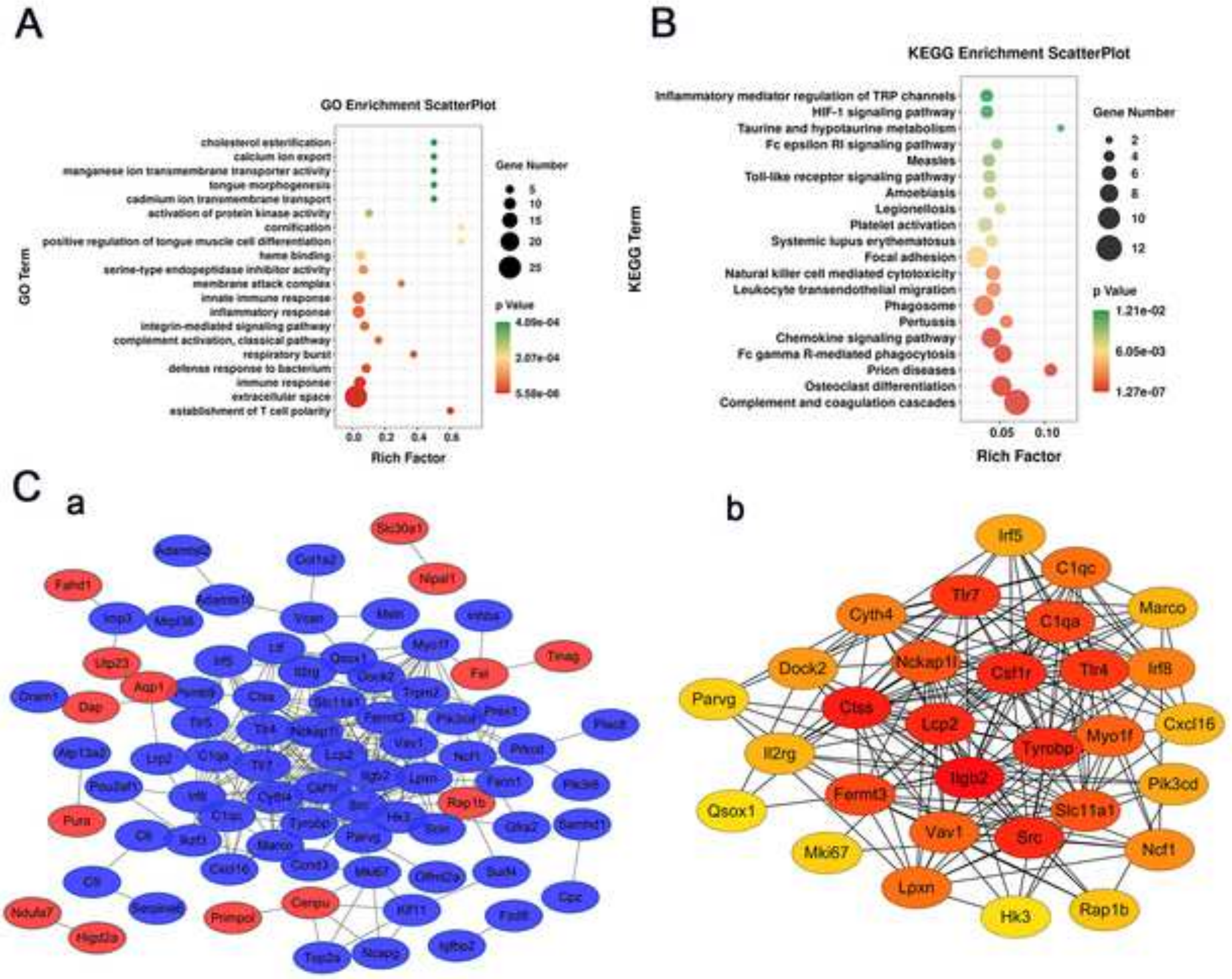

Figure

Click here to access/download;Figure;Fig 5.tif

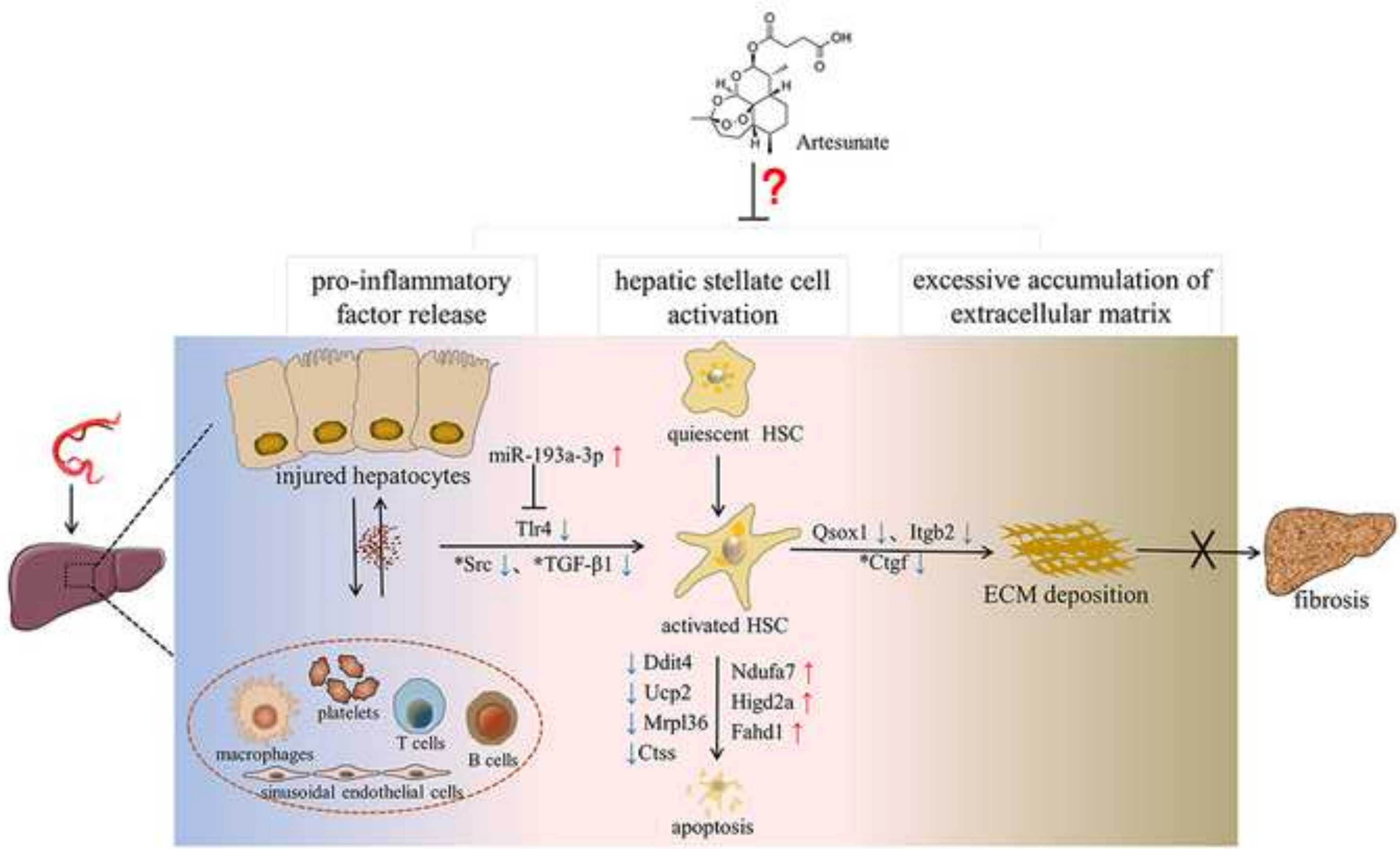

Figure

Click here to access/download
Supporting Information
S1_Fig.tif

Table

Click here to access/download
Supporting Information
S1_Table.doc

Table

Click here to access/download
Supporting Information
S2_Table.doc

Table

Click here to access/download
Supporting Information
S3_Table.doc

Table

Click here to access/download

Supporting Information

S4_Table.doc

Table

Click here to access/download
Supporting Information
S5_Table.docx

Dear editors,

Thank you for giving us the opportunity to submit the revised manuscript entitled "Mining Host Candidate Regulators of Schistosomiasis Liver Fibrosis in Response to Artesunate Therapy through Transcriptomics Approach"

We have incorporated most of the suggestions provided by reviewers. The changes made are highlighted within the manuscript. Please see below, words in blue, for point-by-point responses to reviewers' comments and concerns. All line numbers refer to the revised manuscript file with tracked changes.

To reviewers:

Reviewer #1

Comment 1: Please see the comments I have made on the manuscript, that should be carefully addressed. In several places, additional details regarding materials and methods have been requested. These questions pertain to the design of the study, amount of replication involved, and applicability of some of the bioinformatics approaches. I don't think I have ethical concerns, but would like the level of dosing with ART to be clarified (amount per day, for how many days), and an explanation offered if this is generally considered to be a safe dose, especially at the high level used. The overall effort was hampered by not defining ALL their acronyms clearly - this was a distraction for me.

Response: Thank you for your valuable suggestions. We have carefully revised the manuscript in line with your suggestions and left traces of the modifications. **1)** In the animal experiments, 20 mg/kg/d (low dose) and 40 mg/kg/d (high dose) were administered for 12 weeks. The dose of ART for animal treatment was based on those reported previously which showed an LD50 of 488 mg/kg on acute toxicity of intravenous ART in rats (1). Our previous long-term toxicity study showed that the LD50 of artesunate given orally to rats once daily for 28 d was 150 mg/kg/d and the safe dose was 25 mg/kg/d (2). After conversion, the lethal dose for gerbils is 240 mg/kg/d and the safe dose is 40 mg/kg/d. The US CDC recommends an intravenous dose of 2.4 mg/kg for Malaria for adults. Collectively, our high dose of ART 40mg/kg in gerbil gavage is theoretically relatively safe and effective. **2)** We have consulted with sequencing company professionals who advised that it is the successfully mapped reads which are then used for subsequent analysis rather than the uniquely mapped reads (line 220). **3)** We apologize for not defining all abbreviations which made it difficult for you to review the paper. In the revised version, we have clearly defined all the acronyms and abbreviations on their first occurrence in the full text by writing the term out in full and followed by the abbreviation in brackets.

Comment 2: Again, several requests for additional details have been made on the manuscript. In particular, the figure legends are too brief and need more explanation. Some of the effects noted for ART seem to be marginally significant, even at what seems like high treatment doses. The results of the transcriptome study seem to return an interesting and relevant set of molecules that can plausibly be linked to the fibrosis phenomenon.

Response: Thank you very much for your evaluation of our manuscript. To increase the credibility and clarity of our conclusions, we have improved and supplemented the shortcomings of the experimental details and illustrations (Fig 5). The refinements mainly include: (1) The biochemical parameters including SOD and GPX activity as well as MDA and HYP level were measured. (2) Re-scoring of pathological sections using the Ishak pathology scoring system. (3) We performed WB experiments to determined expression of genes related with fibrosis, such as a-SMA, Collagen

etc.

Comment 3: The conclusions seem to be supported by the data, but some of the limitations noted above with respect to further experimental details will help to clarify their conclusions. Some limitations are mentioned by the authors and this makes sense as they have uncovered new candidate molecules involved in fibrosis, but I have posed some questions along these lines on the manuscript that need to be addressed. There is a broad justification for wanting to better control or reduce fibrosis as it relates to diseases like schistosomiasis. To give their paper more clarity and to potentially increase its significance, it would be helpful for the authors to briefly indicate why fibrosis is bad, but might it be the case that when prevented, it might actually worsen a patient's prospects by facilitating schistosome egg survival and its secretion of harmful substances? It is understood though that exploration of ART and its potential role in controlling fibrosis could have useful applications. I would like the authors to discuss whether the doses required in their study to achieve an effect are practical for human application.

Response: Thank you for the kind and construction suggestions. We have added the following to the discussion with "Schistosomiasis, mostly caused by infection with Schistosoma japonicum, mainly leads to liver fibrosis and advanced disease, such as portal hypertension (3). ART can effectively kill juvenile schistosomulum and has a very good effect of preventing schistosomiasis, so it will not cause the survival and expulsion of eggs (4). However, removal or elimination of the etiological agents may not be sufficient to stop liver fibrosis progression [5]" (line 287-296). The dose of ART for animal treatment was based on those reported previously which showed an LD50 of 488 mg/kg on acute toxicity of intravenous ART in rats (1). Our previous long-term toxicity study showed that the LD50 of artesunate given orally to rats once daily for 28 d was 150 mg/kg/d and the safe dose was 25 mg/kg/d (2). After conversion, the lethal dose for gerbils is 240 mg/kg/d and the safe dose is 40 mg/kg/d. The US CDC recommends an intravenous dose of 2.4 mg/kg for Malaria in adults. Collectively, our high dose of ART 40mg/kg in gerbil gavage is theoretically relatively safe and effective.

Comment 4: Throughout the English is off enough to be a distraction in some key places. Some suggestions are found in my comments but I have not edited it thoroughly for English grammar. The figure legends need to be more thorough.

Response: The paper has been carefully revised by a professional language editing service to improve the grammar and readability. We have added more details in Figure 5 legend in the revised manuscript to illustrate this hypothetical mechanism of ART more clearly. All the figure legends have also been revised correspondingly.

Comment 5: I was intrigued by the fact that ART might have beneficial applications to schistosome-mediated fibrosis so am sympathetic to the overall effort. The presentation was weakened somewhat by the lack of details in methods and explanation of results. Clarity with respect to the doses required to achieve effects and how effectively transcriptomics data from gerbils can be interpreted using a mouse reference genome is particularly needed. Several comments were made directly on the manuscript itself that should be consulted.

Response: Thank you for the valuable comments, we have revised the manuscript according to your kind and construction suggestions. Transcriptomics data used in our manuscript were uploaded to the Sequencing Read Archive of the National Center for Biotechnology Information (NCBI) (Project number PRJNA846574). In fact, the transcriptome data were compared to the gerbil genome (https://www.ncbi.nlm.nih.gov/genome/?term=Mongolian+gerbil) rather than the mouse

genome. We are so sorry for wrongly writting mouse.

Reviewer #2

Yuan and collaborators explored the potential of Artesunate, a well-known antimalarial with anthelminthic potential, to ameliorate liver fibrosis as a result of S japonicum infection in Jirds and the liver tissue analyzed by transcriptomics to unveil possible mechanistic bases for such an effect. ART were reported to inhibit proliferation and induce apoptosis of Hepatic stellate cells in a dose-dependent manner. Levels of HA, ALT and inflammatory cell infiltration were claimed to be decreased after ART administration in vivo with reduction of collagen fibers. Transcriptomic analyses of the liver revealed a set of differentially expressed genes and miRNAs where some hits could be validated for differential expression by qPCR. Interestingly, the authors report on a hub of the 30 most DEGs as mediators of major immune and inflammatory pathways. The authors went on to conclude on the ability of ART to prevent liver fibrosis in Jirds with a provision of candidate genes involved in the regulation of schistosomiasis liver fibrosis in response to ART.

The work is conceptually novel in its proceeding, specifically the use of transcriptomics to reveal candidate regulators of Artesunate-mediated regulation of S. japonicum driven liver fibrosis. The conclusions are appealing but would require considerable caution and additional control experimentation to conclusively support the present claims.

Major comments:

1. How certain are the authors that the PZQ treatment did completely clear the infection? Were worm / egg burden measured after PZQ treatment and at the end of the subsequent ART treatment to ensure that the reduction in the progression of liver fibrosis observed are as a result of the anti-fibrotic potential of ART and not mostly due to its additional antiparasitic potential on S japonicum? (**Xiao et al., Chin Med J (Engl). 1996 Apr;109(4):272-5; li et al, Acta Trop. Nov-Dec 2005;96(2-3):184-90.**).

Response: Thank you for the kind and construction comments. We have read through the above two articles, in which the worm and egg burden were measured. The study showed that PZQ has good efficacy with oral administration of praziquantel at a single dose of 400 mg/kg resulting in worm reduction rates of 88-100% in mice (5). Through dose conversion, it can also be inferred that the dose we used (75 mg/kg for 3 days) can completely reduce adult worms. We examined histopathology at 4 weeks after PZQ administration and found no live adult worms and eggs under ordinary light microscopy. Since the supplementary worm and egg burden experiment requires collection of positive nails, which requires a long experimental period, we plan to investigate it in future.

2. Figure 2B. HA and ALT levels should also be compared between the ART groups and the Model groups to reflect true reduction of these factors by ART treatment specifically. As it stands, HA and ALT levels do not appear to decay in ART groups when compared to Model groups arguing against a liver protecting effect of the ART treatment.

Response: The clinical diagnostic value of HA and ALT serological tests is affected by many factors, including surgical technique, tissue damage, and the post-operative environment. Therefore, to increase the confidence of the results, we added other fibrosis-related quantitative indicators such as HYP level in liver tissue. In addition, pathological diagnosis is the gold standard diagnostic tool for fibrosis, and thus to evaluate the dynamic changes in the quality of fibrosis before and after fibrosis treatment. The Ishak scoring system was utilized to evaluate the degree of liver inflammation and fibrosis in this study (6).

3. Figure 2C is highly qualitative, thus suggestive, and would benefit from a quantitative assay of measurement of tissue fibrosis such as hydroxyproline quantification. Moreover here, the staining scores do not reflect an overall reduction in the Model group when compared to the ART (after administration) group arguing strongly against an anti-fibrotic effect of ART in this study (critical! As this is the basis for the subsequent transcriptomic screening). One would have expected the ART groups after administration to be lower than the Model group even before administration. The continued increase of the fibrosis score in the Model group after administration when compared to the model group before administration does suggest an inability of the prior PZQ treatment to completely clear the schistosoma infection and would support that all effects observed with ART treatment might result from an anti-parasitic rather than anti-fibrotic action.

Response: Thank you for the valuable comments and suggestions. We have modified the manuscript accordingly. In addition, we provide the followings answers to your comments: **1)** To make the results more convincing, we have added data of fibrosis-related biochemical quantitative indicators such as HYP and liver oxidative damage-related indicators such as SOD and MDA levels in Figure 2B. **2)** The traditional evaluation system used in the original manuscript mainly focus on the severity rather than the dynamic changes in fibrosis. Therefore, re re-scored the pathological sections using the Ishak scoring system before and after ART treatment (6). **3)** We performed histopathological examination prior to ART administration and found no adult worms and live eggs, proving that PZQ completely dewormed the animal. Additionally, PZQ has been approved for the treatment of schistosomiasis. Oral administration of PZQ at a single dose of 400 mg/kg resulted in worm reduction rates of 88-100% in mice (5), however, removal or elimination of the etiological agents may not be sufficient to stop liver fibrosis progression (7). Studies have shown that ART is effective against juvenile schistosomulum, and that schistosome eggs survive in the body for only about 21 days under the influence of the host immune system (4). PZQ did not reduce fibrosis in the MOD group, which indicated that the reduction of fibrosis in the ART group was due to its anti-fibrotic effect rather than its antimalarial effect.

4. Why were markers of fibrosis not tested by qPCR as well? i.e. a-SMA, Collagen etc… to support the anti-fibrotic claim of ART treatment in this particular study.

Response: Thank you very much for the comments and suggestions. To make the results and conclusions more convincing, we performed WB experiments to determined expression of these genes, such as a-SMA, Collagen etc. And the results of the western blot experiment we performed were relatively consistent with the sequencing results.

Minor comments:

1. Language is to be carefully checked throughout the manuscript for grammatical mistakes and typos eg. line 90 '…in the fluorescence microscope' should read '…under the fluorescence microscope'; Line 121:' Total RNA was isolated form…' should read ' total RNA was isolated from…'

Response: We apologize for the language problems. The paper has been carefully revised by a professional language editing service to improve the grammar and readability.

2. Caution should be made on exaggerated statements: 'We developed liver fibrosis model in Mongolian gerbil mimicking human infection with S. japonicum cercariae'. This model is well known and merely reproduced by the authors. Eg. Liang et al., Int J Parasitol. 1983 Dec;13(6):531-8. doi: 10.1016/s0020-7519(83)80024-1.

Response: Thank you for pointing this out. We have revised the manuscript and left traces in the text and attached reference.

3. How do the identified factors relate to previously reported factors in the literature using other omics approaches following ART treatment of S japonicum-infected rodents (Kong et al., Mol Biosyst. 2015 May;11(5):1400-9. doi: 10.1039/c5mb00074b.) Is a consensus observable here. Harmonize the referencing style to that of Plos NTDs throughout the manuscript eg. Line 294 :' … that Src is a potential target of ART to liver fibrosis [23](Berkoz et al. 2021).

Response: Thank you for the questions. The subjects of the two studies were different. The study, published in 2015, aimed to investigate the effects of ART on schistosoma itself, while the purpose of our current paper is to explore the mechanism of ART on host fibrosis caused by schistosomiasis infection. We have harmonized the reference style with the citation style of Plos NTDs throughout the manuscript.

Reviewer #3

Comment 1: The expression trend of qPCR targeted Ctss, Mgp, Vim, Ctgf, Cd74, Chi3l1 was consistent with the sequencing results. These genes need to be validated by WB.

Response: We appreciate your kind suggestions. Due to the long purchase time of some antibodies, we supplement the WB experiment of these genes using existing antibodies (Fig 3E).

Comment 2: A scale bar or magnification should be indicated on the picture.

Response: Thank you for this suggestion. We have re-selected typical pathology pictures and added scale bar on the picture and magnification in the corresponding legend (Fig 1 and Fig 2).

We hereby resubmit the revised manuscript and hope that these corrections would be satisfactory. Please feel free to contact us with any questions and we look forward to your decision.

Reference:

1. Li Q, Xie LH, Johnson TO, Si Y, Haeberle AS, Weina PJ. Toxicity evaluation of artesunate and artelinate in Plasmodium berghei-infected and uninfected rats. Trans R Soc Trop Med Hyg. 2007;101(2):104-12.
2. Guo Y WL, Xu PS, Yang MJ, Fan JT, Li SW. Safety assessment for long-term oral treatment with artesunate in rats. Zhongguo Xue Xi Chong Bing Fang Zhi Za Zhi. 1998;12(1):17-9. (in Chinese).
3. Gryseels B, Polman K, Clerinx J, Kestens L. Human schistosomiasis. Lancet. 2006;368(9541):1106-18.
4. Gouveia MJ, Brindley PJ, Gartner F, Costa J, Vale N. Drug Repurposing for Schistosomiasis: Combinations of Drugs or Biomolecules. Pharmaceuticals (Basel). 2018;11(1).
5. Xiao SH, Sun J, Chen MG. Pharmacological and immunological effects of praziquantel against Schistosoma japonicum: a scoping review of experimental studies. Infect Dis Poverty. 2018;7(1):9.
6. Ishak K, Baptista A, Bianchi L, Callea F, De Groote J, Gudat F, et al. Histological grading and staging of chronic hepatitis. J Hepatol. 1995;22(6):696-9.
7. Wang FD, Zhou J, Chen EQ. Molecular Mechanisms and Potential New Therapeutic Drugs for Liver Fibrosis. Front Pharmacol. 2022;13:787748.

Click here to access/download
Revised Article with Changes Highlighted
Regulators of Schistosomiasis Liver Fibrosis to Artesunate.doc
