# Supplementary material for: Mining host candidate regulators of schistosomiasis-induced liver fibrosis in response to artesunate therapy through transcriptomics approach
Source: PLoS Negl Trop Dis. 2023 Sep 29;17(9):e0011626. doi: 10.1371/journal.pntd.0011626 (PMC10566724; doi:10.1371/journal.pntd.0011626)
Supplement: Attachment — Submitted filename: Response letter.docx [file pntd.0011626.s008.docx]

Dear editors,

Thank you for giving us the opportunity to submit the revised manuscript entitled “Mining Host Candidate Regulators of Schistosomiasis Liver Fibrosis in Response to Artesunate Therapy through Transcriptomics Approach”

We have incorporated most of the suggestions provided by reviewers. The changes made are highlighted within the manuscript. Please see below, words in blue, for point-by-point responses to reviewers’ comments and concerns. All line numbers refer to the revised manuscript file with tracked changes.

**To reviewers:**

**Reviewer #1**

**Comment 1**: Please see the comments I have made on the manuscript, that should be carefully addressed. In several places, additional details regarding materials and methods have been requested. These questions pertain to the design of the study, amount of replication involved, and applicability of some of the bioinformatics approaches. I don't think I have ethical concerns, but would like the level of dosing with ART to be clarified (amount per day, for how many days), and an explanation offered if this is generally considered to be a safe dose, especially at the high level used. The overall effort was hampered by not defining ALL their acronyms clearly - this was a distraction for me.

**Response:** Thank you for your valuable suggestions. We have carefully revised the manuscript in line with your suggestions and left traces of the modifications. **1)** In the animal experiments, 20 mg/kg/d (low dose) and 40 mg/kg/d (high dose) were administered for 12 weeks. The dose of ART for animal treatment was based on those reported previously which showed an LD50 of 488 mg/kg on acute toxicity of intravenous ART in rats (1). Our previous long-term toxicity study showed that the LD50 of artesunate given orally to rats once daily for 28 d was 150 mg/kg/d and the safe dose was 25 mg/kg/d (2). After conversion, the lethal dose for gerbils is 240 mg/kg/d and the safe dose is 40 mg/kg/d. The US CDC recommends an intravenous dose of 2.4 mg/kg for Malaria for adults.

Collectively, our high dose of ART 40mg/kg in gerbil gavage is theoretically relatively safe and effective. **2)** We have consulted with sequencing company professionals who advised that it is the successfully mapped reads which are then used for subsequent analysis rather than the uniquely mapped reads (line 220). **3)** We apologize for not defining all abbreviations which made it difficult for you to review the paper. In the revised version, we have clearly defined all the acronyms and abbreviations on their first occurrence in the full text by writing the term out in full and followed by the abbreviation in brackets.

**Comment 2:** Again, several requests for additional details have been made on the manuscript. In particular, the figure legends are too brief and need more explanation. Some of the effects noted for ART seem to be marginally significant, even at what seems like high treatment doses. The results of the transcriptome study seem to return an interesting and relevant set of molecules that can plausibly be linked to the fibrosis phenomenon.

**Response:** Thank you very much for your evaluation of our manuscript. To increase the credibility and clarity of our conclusions, we have improved and supplemented the shortcomings of the experimental details and illustrations (Fig 5). The refinements mainly include: (1) The biochemical parameters including SOD and GPX activity as well as MDA and HYP level were measured. (2) Re-scoring of pathological sections using the Ishak pathology scoring system. (3) We performed WB experiments to determined expression of genes related with fibrosis, such as a-SMA, Collagen etc.

**Comment 3:** The conclusions seem to be supported by the data, but some of the limitations noted above with respect to further experimental details will help to clarify their conclusions. Some limitations are mentioned by the authors and this makes sense as they have uncovered new candidate molecules involved in fibrosis, but I have posed some questions along these lines on the manuscript that need to be addressed. There is a broad justification for wanting to better control or reduce fibrosis as it relates to diseases like schistosomiasis. To give their paper more clarity and to potentially increase its significance, it would be helpful for the authors to briefly indicate why fibrosis is bad, but might it be the case that when prevented, it might actually worsen a patient's prospects by facilitating schistosome egg survival and its secretion of harmful substances? It is understood though that exploration of ART and its potential role in controlling fibrosis could have useful applications. I would like the authors to discuss whether the doses required in their study to achieve an effect are practical for human application.

**Response:** Thank you for the kind and construction suggestions. We have added the following to the discussion with “Schistosomiasis, mostly caused by infection with Schistosoma japonicum, mainly leads to liver fibrosis and advanced disease, such as portal hypertension (3). ART can effectively kill juvenile schistosomulum and has a very good effect of preventing schistosomiasis, so it will not cause the survival and expulsion of eggs (4). However, removal or elimination of the etiological agents may not be sufficient to stop liver fibrosis progression [5]” (line 287-296). The dose of ART for animal treatment was based on those reported previously which showed an LD50 of 488 mg/kg on acute toxicity of intravenous ART in rats (1). Our previous long-term toxicity study showed that the LD50 of artesunate given orally to rats once daily for 28 d was 150 mg/kg/d and the safe dose was 25 mg/kg/d (2). After conversion, the lethal dose for gerbils is 240 mg/kg/d and the safe dose is 40 mg/kg/d. The US CDC recommends an intravenous dose of 2.4 mg/kg for Malaria in adults. Collectively, our high dose of ART 40mg/kg in gerbil gavage is theoretically relatively safe and effective.

**Comment 4:** Throughout the English is off enough to be a distraction in some key places. Some suggestions are found in my comments but I have not edited it thoroughly for English grammar. The figure legends need to be more thorough.

**Response:** The paper has been carefully revised by a professional language editing service to improve the grammar and readability. We have added more details in Figure 5 legend in the revised manuscript to illustrate this hypothetical mechanism of ART more clearly. All the figure legends have also been revised correspondingly.

**Comment 5:** I was intrigued by the fact that ART might have beneficial applications to schistosome-mediated fibrosis so am sympathetic to the overall effort. The presentation was weakened somewhat by the lack of details in methods and explanation of results. Clarity with respect to the doses required to achieve effects and how effectively transcriptomics data from gerbils can be interpreted using a mouse reference genome is particularly needed. Several comments were made directly on the manuscript itself that should be consulted.

**Response:** Thank you for the valuable comments, we have revised the manuscript according to your kind and construction suggestions. Transcriptomics data used in our manuscript were uploaded to the Sequencing Read Archive of the National Center for Biotechnology Information (NCBI) (Project number PRJNA846574). In fact, the transcriptome data were compared to the gerbil genome (https://www.ncbi.nlm.nih.gov/genome/?term=Mongolian+gerbil) rather than the mouse genome. We are so sorry for wrongly writting mouse.

**Reviewer #2**

Yuan and collaborators explored the potential of Artesunate, a well-known antimalarial with anthelminthic potential, to ameliorate liver fibrosis as a result of S japonicum infection in Jirds and the liver tissue analyzed by transcriptomics to unveil possible mechanistic bases for such an effect.

ART were reported to inhibit proliferation and induce apoptosis of Hepatic stellate cells in a dose-dependent manner. Levels of HA, ALT and inflammatory cell infiltration were claimed to be decreased after ART administration in vivo with reduction of collagen fibers. Transcriptomic analyses of the liver revealed a set of differentially expressed genes and miRNAs where some hits could be validated for differential expression by qPCR. Interestingly, the authors report on a hub of the 30 most DEGs as mediators of major immune and inflammatory pathways. The authors went on to conclude on the ability of ART to prevent liver fibrosis in Jirds with a provision of candidate genes involved in the regulation of schistosomiasis liver fibrosis in response to ART.

The work is conceptually novel in its proceeding, specifically the use of transcriptomics to reveal candidate regulators of Artesunate-mediated regulation of S. japonicum driven liver fibrosis. The conclusions are appealing but would require considerable caution and additional control experimentation to conclusively support the present claims.

Major comments:

1. How certain are the authors that the PZQ treatment did completely clear the infection? Were worm / egg burden measured after PZQ treatment and at the end of the subsequent ART treatment to ensure that the reduction in the progression of liver fibrosis observed are as a result of the anti-fibrotic potential of ART and not mostly due to its additional antiparasitic potential on S japonicum? (**Xiao et al., Chin Med J (Engl). 1996 Apr;109(4):272-5; li et al, Acta Trop. Nov-Dec 2005;96(2-3):184-90.**).

**Response:** Thank you for the kind and construction comments. We have read through the above two articles, in which the worm and egg burden were measured. The study showed that PZQ has good efficacy with oral administration of praziquantel at a single dose of 400 mg/kg resulting in worm reduction rates of 88-100% in mice (5). Through dose conversion, it can also be inferred that the dose we used (75 mg/kg for 3 days) can completely reduce adult worms. We examined histopathology at 4 weeks after PZQ administration and found no live adult worms and eggs under ordinary light microscopy. Since the supplementary worm and egg burden experiment requires collection of positive nails, which requires a long experimental period, we plan to investigate it in future.

2. Figure 2B. HA and ALT levels should also be compared between the ART groups and the Model groups to reflect true reduction of these factors by ART treatment specifically. As it stands, HA and ALT levels do not appear to decay in ART groups when compared to Model groups arguing against a liver protecting effect of the ART treatment.

**Response:** The clinical diagnostic value of HA and ALT serological tests is affected by many factors, including surgical technique, tissue damage, and the post-operative environment. Therefore, to increase the confidence of the results, we added other fibrosis-related quantitative indicators such as HYP level in liver tissue. In addition, pathological diagnosis is the gold standard diagnostic tool for fibrosis, and thus to evaluate the dynamic changes in the quality of fibrosis before and after fibrosis treatment. The Ishak scoring system was utilized to evaluate the degree of liver inflammation and fibrosis in this study (6).

3. Figure 2C is highly qualitative, thus suggestive, and would benefit from a quantitative assay of measurement of tissue fibrosis such as hydroxyproline quantification. Moreover here, the staining scores do not reflect an overall reduction in the Model group when compared to the ART (after administration) group arguing strongly against an anti-fibrotic effect of ART in this study (critical! As this is the basis for the subsequent transcriptomic screening). One would have expected the ART groups after administration to be lower than the Model group even before administration. The continued increase of the fibrosis score in the Model group after administration when compared to the model group before administration does suggest an inability of the prior PZQ treatment to completely clear the schistosoma infection and would support that all effects observed with ART treatment might result from an anti-parasitic rather than anti-fibrotic action.

**Response:** Thank you for the valuable comments and suggestions. We have modified the manuscript accordingly. In addition, we provide the followings answers to your comments: **1)** To make the results more convincing, we have added data of fibrosis-related biochemical quantitative indicators such as HYP and liver oxidative damage-related indicators such as SOD and MDA levels in Figure 2B. **2)** The traditional evaluation system used in the original manuscript mainly focus on the severity rather than the dynamic changes in fibrosis. Therefore, re re-scored the pathological sections using the Ishak scoring system before and after ART treatment (6). **3)** We performed histopathological examination prior to ART administration and found no adult worms and live eggs, proving that PZQ completely dewormed the animal. Additionally, PZQ has been approved for the treatment of schistosomiasis. Oral administration of PZQ at a single dose of 400 mg/kg resulted in worm reduction rates of 88-100% in mice (5), however, removal or elimination of the etiological agents may not be sufficient to stop liver fibrosis progression (7). Studies have shown that ART is effective against juvenile schistosomulum, and that schistosome eggs survive in the body for only about 21 days under the influence of the host immune system (4). PZQ did not reduce fibrosis in the MOD group, which indicated that the reduction of fibrosis in the ART group was due to its anti-fibrotic effect rather than its antimalarial effect.

4. Why were markers of fibrosis not tested by qPCR as well? i.e. a-SMA, Collagen etc… to support the anti-fibrotic claim of ART treatment in this particular study.

**Response:** Thank you very much for the comments and suggestions. To make the results and conclusions more convincing, we performed WB experiments to determined expression of these genes, such as a-SMA, Collagen etc. And the results of the western blot experiment we performed were relatively consistent with the sequencing results.

**Minor comments**：

1. Language is to be carefully checked throughout the manuscript for grammatical mistakes and typos eg. line 90 ‘…in the fluorescence microscope’ should read ‘…under the fluorescence microscope’; Line 121:’ Total RNA was isolated form…’ should read ‘ total RNA was isolated from…’

**Response:** We apologize for the language problems. The paper has been carefully revised by a professional language editing service to improve the grammar and readability.

2. Caution should be made on exaggerated statements: ‘We developed liver fibrosis model in Mongolian gerbil mimicking human infection with S. japonicum cercariae’. This model is well known and merely reproduced by the authors. Eg. Liang et al., Int J Parasitol. 1983 Dec;13(6):531-8. doi: 10.1016/s0020-7519(83)80024-1.

**Response:** Thank you for pointing this out. We have revised the manuscript and left traces in the text and attached reference.

3. How do the identified factors relate to previously reported factors in the literature using other omics approaches following ART treatment of S japonicum-infected rodents (Kong et al., Mol Biosyst. 2015 May;11(5):1400-9. doi: 10.1039/c5mb00074b.) Is a consensus observable here.

Harmonize the referencing style to that of Plos NTDs throughout the manuscript eg. Line 294 :’… that Src is a potential target of ART to liver fibrosis [23](Berkoz et al. 2021).

**Response:** Thank you for the questions. The subjects of the two studies were different. The study, published in 2015, aimed to investigate the effects of ART on schistosoma itself, while the purpose of our current paper is to explore the mechanism of ART on host fibrosis caused by schistosomiasis infection. We have harmonized the reference style with the citation style of Plos NTDs throughout the manuscript.

**Reviewer #3**

**Comment 1:** The expression trend of qPCR targeted Ctss, Mgp, Vim, Ctgf, Cd74, Chi3l1 was consistent with the sequencing results. These genes need to be validated by WB.

**Response:** We appreciate your kind suggestions. Due to the long purchase time of some antibodies, we supplement the WB experiment of these genes using existing antibodies (Fig 3E).

**Comment 2:** A scale bar or magnification should be indicated on the picture.

**Response:** Thank you for this suggestion. We have re-selected typical pathology pictures and added scale bar on the picture and magnification in the corresponding legend (Fig 1 and Fig 2).

We hereby resubmit the revised manuscript and hope that these corrections would be satisfactory. Please feel free to contact us with any questions and we look forward to your decision.

**Reference:**

1.	Li Q, Xie LH, Johnson TO, Si Y, Haeberle AS, Weina PJ. Toxicity evaluation of artesunate and artelinate in Plasmodium berghei-infected and uninfected rats. Trans R Soc Trop Med Hyg. 2007;101(2):104-12.

2.	Guo Y WL, Xu PS, Yang MJ, Fan JT, Li SW. Safety assessment for long-term oral treatment with artesunate in rats. Zhongguo Xue Xi Chong Bing Fang Zhi Za Zhi. 1998;12(1):17-9. (in Chinese).

3.	Gryseels B, Polman K, Clerinx J, Kestens L. Human schistosomiasis. Lancet. 2006;368(9541):1106-18.

4.	Gouveia MJ, Brindley PJ, Gartner F, Costa J, Vale N. Drug Repurposing for Schistosomiasis: Combinations of Drugs or Biomolecules. Pharmaceuticals (Basel). 2018;11(1).

5.	Xiao SH, Sun J, Chen MG. Pharmacological and immunological effects of praziquantel against Schistosoma japonicum: a scoping review of experimental studies. Infect Dis Poverty. 2018;7(1):9.

6.	Ishak K, Baptista A, Bianchi L, Callea F, De Groote J, Gudat F, et al. Histological grading and staging of chronic hepatitis. J Hepatol. 1995;22(6):696-9.

7.	Wang FD, Zhou J, Chen EQ. Molecular Mechanisms and Potential New Therapeutic Drugs for Liver Fibrosis. Front Pharmacol. 2022;13:787748.
