# Supplementary material for: Mining host candidate regulators of schistosomiasis-induced liver fibrosis in response to artesunate therapy through transcriptomics approach
Source: PLoS Negl Trop Dis. 2023 Sep 29;17(9):e0011626. doi: 10.1371/journal.pntd.0011626 (PMC10566724; doi:10.1371/journal.pntd.0011626)
Supplement: Attachment — Submitted filename: Response letter.docx [file pntd.0011626.s010.docx]

**Dear editors**,

Thank you very much for giving us the opportunity to submit the revised manuscript entitled “Mining Host Candidate Regulators of Schistosomiasis Liver Fibrosis in Response to Artesunate Therapy through Transcriptomics Approach”

We have incorporated most of the suggestions provided by reviewers. The changes made are highlighted within the manuscript. Please see below, words in blue, for point-by-point responses to reviewers’ comments and concerns.

**Reply to Reviewer #1 as follows:**

Thank you very much for your attentive and warm comments, we have made corrections to the writing errors in the article, and the Figure legends of Fig 1, Fig 2 and Fig 3 have been explained in detail. We highlight all the changes we made in the article. In addition, the questions that need to be explained to you are shown in the reply below.

**Comments:**

1. Line 72: ART also has a direct negative effect on schistosomes too, so fewer schistosomes, less fibrosis.

**Response:** There is a report showed that after curing for over 20 years, some schistosomiasis patients developed advanced schistosomiasis including liver fibrosis, spleen enlargement, and portal hypertension which have not been eradicated [1].

2. Line 144: referring to 2 livers per treatment group? Is this enough?

**Response:** The serological index detection and histopathological observation, which are based on individual observation. Sequencing tissues are selected from individuals with obvious differences in serological indicators and histopathology, therefore two samples per treatment are enough.

3. Line 204: Where are the results regarding viability as determined by measuring the absorbance at 450 nm? This seems to have disappeared from the paper.

**Response:** Figure 1A is the state of HSC cells after different concentrations of ART treatment under light microscopy, and Figure 1B is the number of viable cells tested at 450 nm absorbance and the histogram obtained by calculating cell viability, that is, Figure 1B is the result of measuring the absorbance value of 450 nm.

**Reply to Reviewer 2 as follows:**

Thank you very much for your critical and constructive comments which are very helpful for the improvement of our study and the strong support of our conclusions. Yet, because we didn’t get enough positive snails this time, the infection experiment was interrupted. Due to the long infection cycle, this experiment could not be carried out again this time. In the future, if there is an opportunity to obtain enough positive snails, we will further verify it. We provide some previous researches to indirectly validate our conclusions for your concerns about the missing verification of the complete clearance infection by PZQ and direct anti-fibrotic potential of artesunate.

**Response:**

1. Our PZQ treatment dose can kill eggs almost completely as discussed in the discussion section.
2. ART is not an ideal drug on eggs, so it is not very convincing that ART indirectly exert anti-fibrotic effects through its direct pharmacological effect on eggs.
3. It is reported that 75 original schistosomiasis cases cured for over 20 years still developed into advanced schistosomiasis and liver fibrosis has not been eradicated [1].
4. ART has shown significant anti-fibrosis effects in various kinds of fibrosis models, which shows that ART has a certain efficacy in fibrosis regression [2].

We believe what your comments point out are a limitation of our research, which we have discussed them in detail in the discussion so that the design can be more in-depth and refined in the future.

[1] Hua H, Yin A, Xu M, Zhou Z, You L, Guo H. Advanced schistosomiasis reappeared after curing seemingly being cured for over 20 years and without known history of reexposure to Schistosoma japonicum. Parasitol Res. 2015 Sep;114(9):3535-8. doi: 10.1007/s00436-015-4616-7. Epub 2015 Jul 15. PMID: 26174487.

[2] Wang Y, Wang Y, You F, Xue J. Novel use for old drugs: The emerging role of artemisinin and its derivatives in fibrosis. Pharmacol Res. 2020 Jul;157:104829. doi: 10.1016/j.phrs.2020.104829. Epub 2020 Apr 30. PMID: 32360483.
